# Supplementary material for: Comparison of Magnetic Resonance Imaging and Serum Biomarkers for Detection of Human Pluripotent Stem Cell-Derived Teratomas
Source: Stem Cell Reports. 2016 Jan 14;6(2):176–87. doi: 10.1016/j.stemcr.2015.12.008 (PMC4750097; doi:10.1016/j.stemcr.2015.12.008)
Supplement: Document S1. Supplemental Experimental Procedure, Figures S1–S6, and Tables S1–S5 [file mmc1.pdf]

Stem Cell Reports, Volume *6*

Supplemental Information

Comparison of Magnetic Resonance Imaging and Serum Biomarkers for Detection of Human Pluripotent Stem Cell-Derived Teratomas

Johannes Riegler, Antje Ebert, Xulei Qin, Qi Shen, Mouer Wang, Mohamed Ameen, Kazuki Kodo, Sang-Ging Ong, Won Hee Lee, Grace Lee, Evgenios Neofytou, Joseph D. Gold, Andrew J. Connolly, and Joseph C. Wu

SUPPLEMENTAL FIGURES

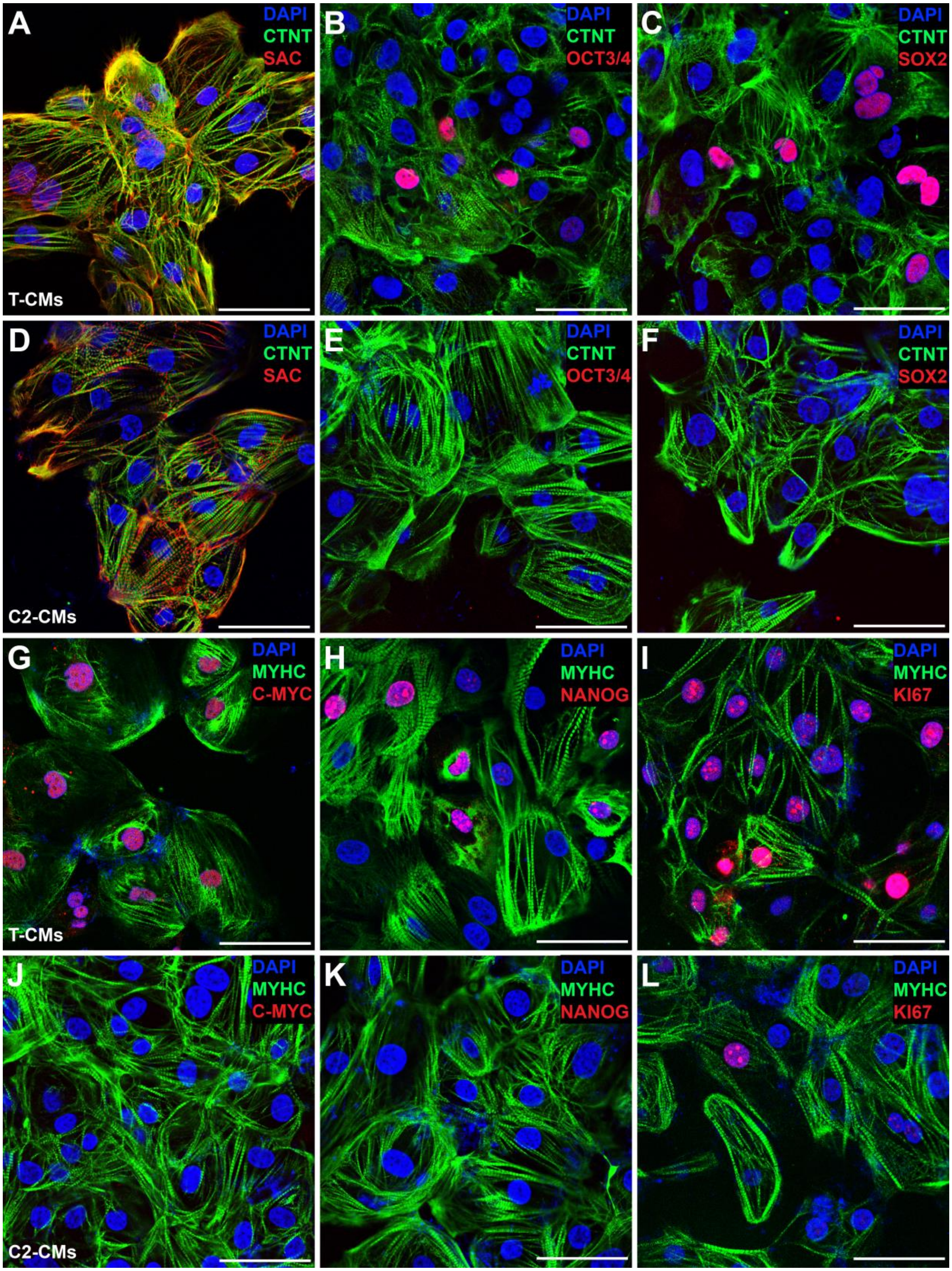

Supplemental Figure S1

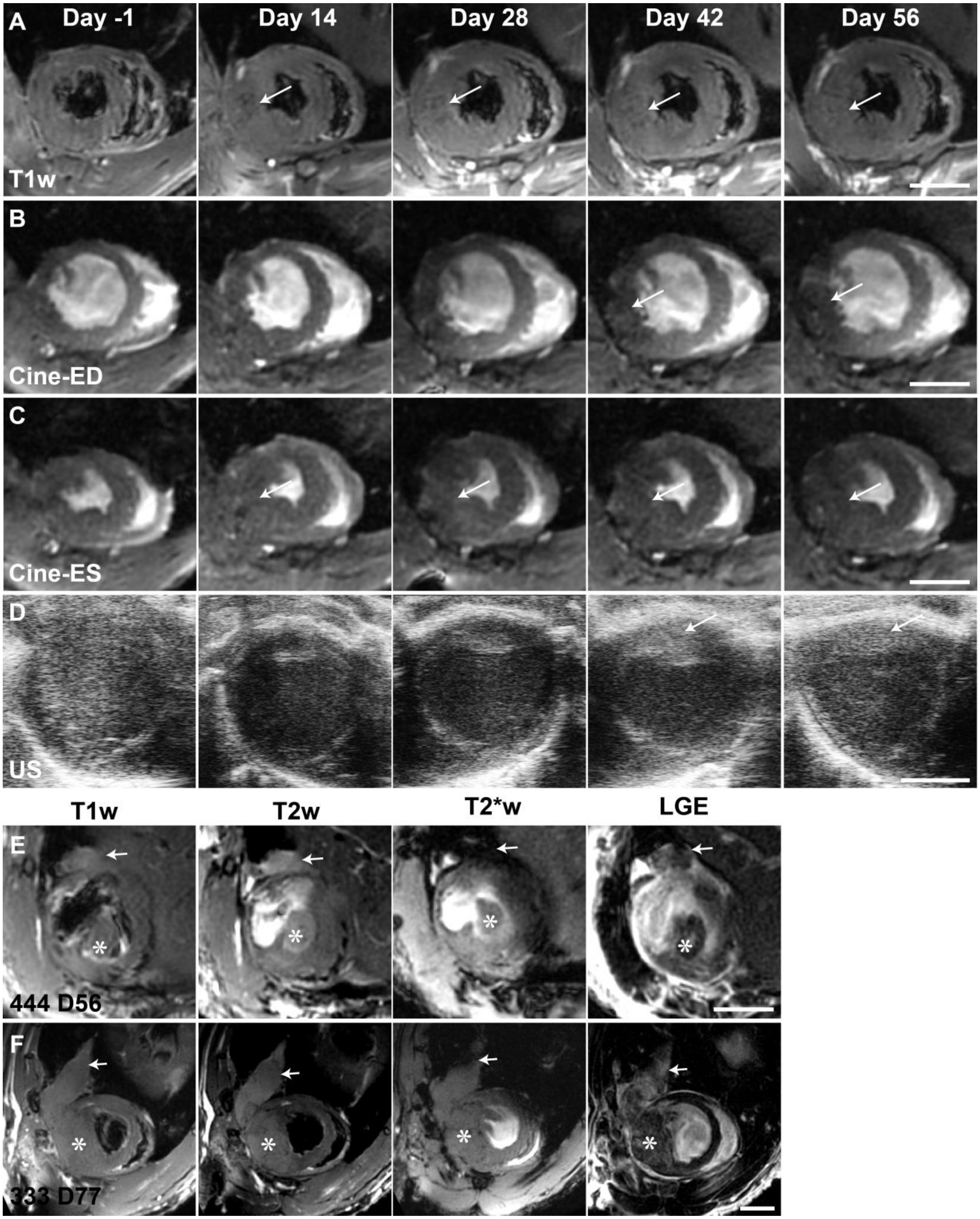

Supplemental Figure S2

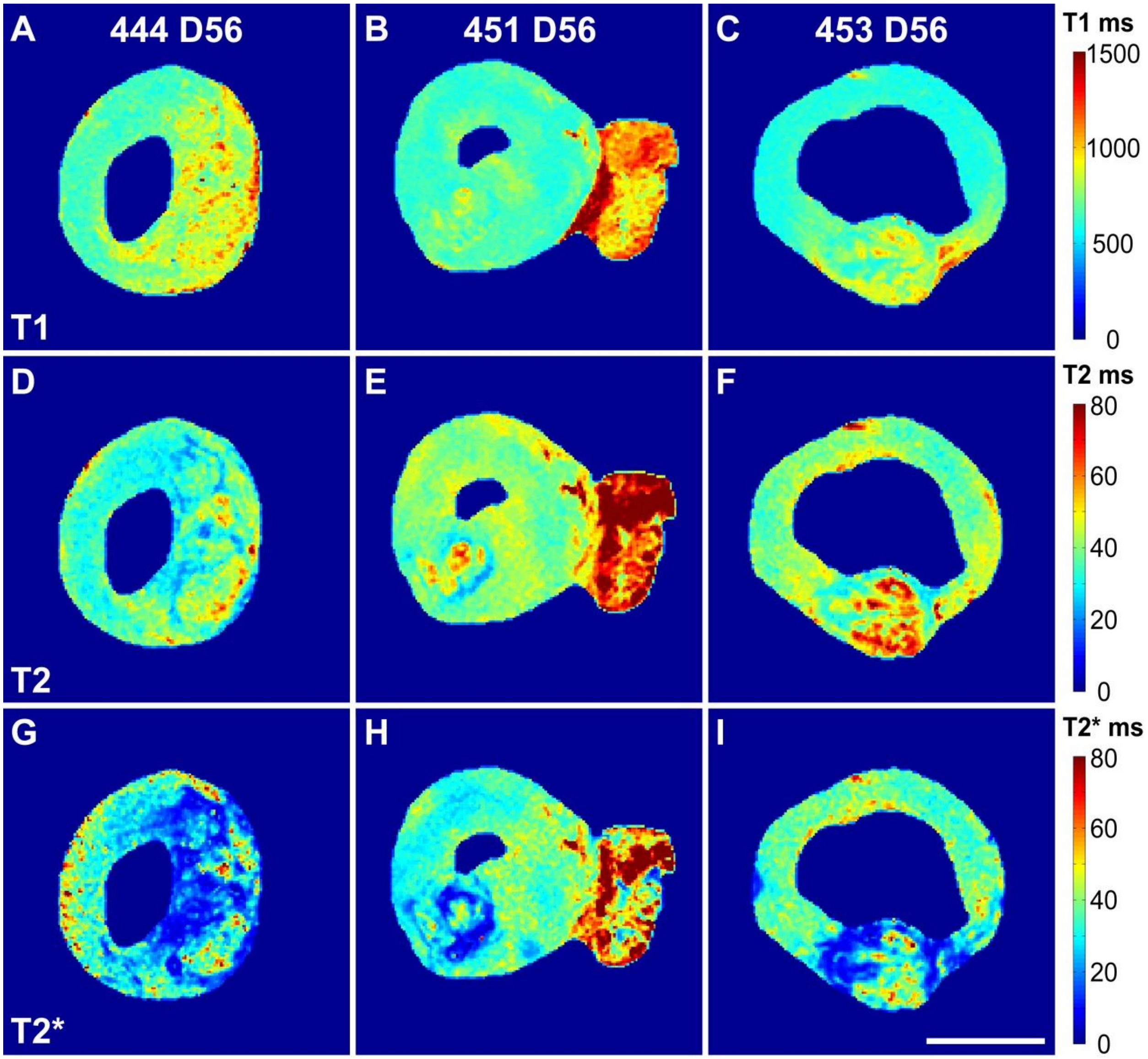

Supplemental Figure S3

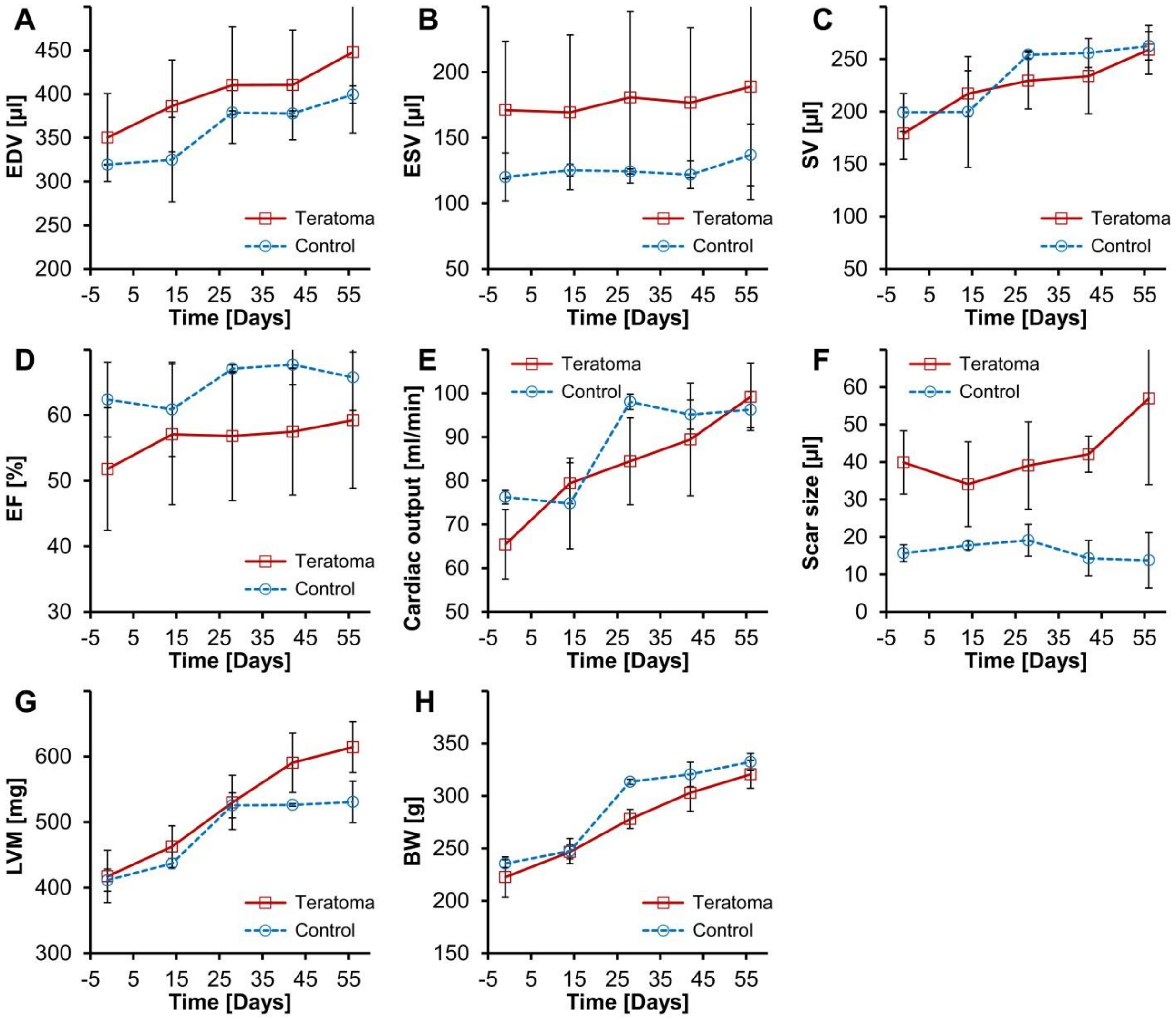

Supplemental Figure S4

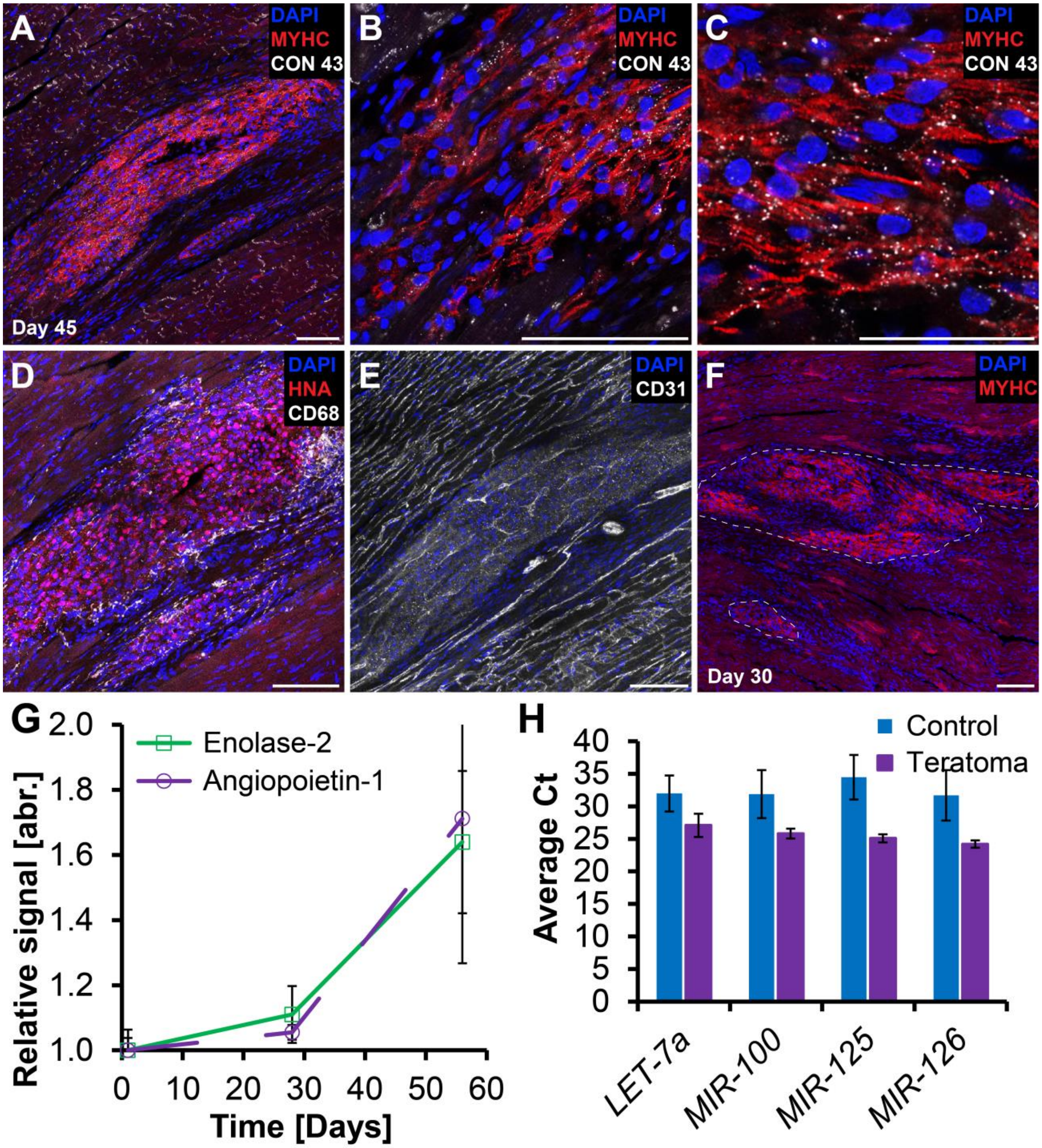

Supplemental Figure S5

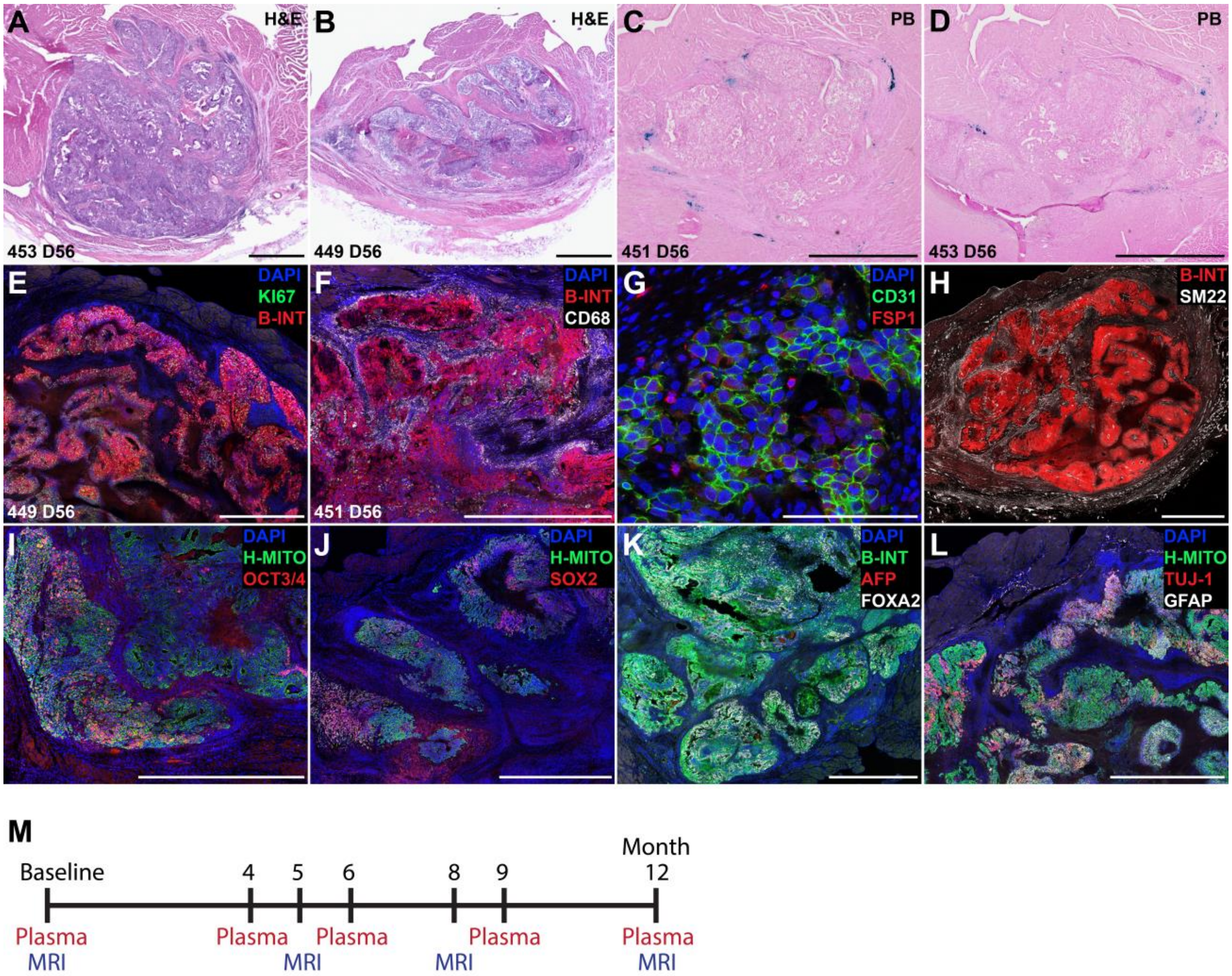

Supplemental Figure S6

SUPPLEMENTAL FIGURE LEGENDS

Supplemental Figure S1: Lentiviral-derived iPSC-CMs showed continued expression of pluripotency markers in contrast to Sendai virus-derived iPSC-CMs. (A-C) Human CMs from lentiviral iPSC line T showed normal sarcomeric structures (cardiac troponin T: CTNT; sarcomeric alpha actinin: SAC), but continued to express OCT3/4 and SOX2. **(D-F)** CMs from control iPSCs (C2) which were generated via a non-integrating method (Sendai virus) displayed normal sarcomeric structures and stained negative for pluripotency markers OCT3/4 and SOX2. **(G-I)** Almost all CMs from the lentiviral line T stained positive for the cell cycle marker C-MYC and about half of the CMs stained positive for NANOG. CMs from this cell line were highly proliferative with all nuclei staining positive for KI67. **(J-L)** CMs from a Sendai virus reprogrammed control iPSC line (C2) did not contain any C-MYC or NANOG positive cells. Only a small number of CM nuclei stained positive for KI67 indicating limited proliferation (representative images from two independent differentiation lots). Scale bars: 50 µm.

Supplemental Figure S2: Serial MRI using T_1w and cine acquisitions as well as ultrasound depicting teratoma growth. (A) Representative T_1-weighted (T1w) images of a rat heart one day before and 2, 4, 6, and 8 weeks after cell delivery. T_1 differences between teratoma and normal myocardium were insufficient to generate enough contrast. The teratoma could only be detected indirectly via increased myocardial wall thickness (white arrows are pointing to teratomas). **(B,C)** End-diastolic (ED) and end-systolic (ES) cine images from the same animal. Similar to T_1w images, teratomas could only be detected indirectly via increased myocardial wall thickness. **(D)** Teratomas could be detected via increased myocardial wall thickness on ultrasound images once they had reached a size of >50 mm^3 (white arrows are pointing to teratomas). **(E-F)** T_1-weighted (T1w), T_2-weighted (T2w), T_2*-weighted (T2*w), and late gadolinium enhancement (LGE) images from two teratomas at 56 and 77 days that had spread to the surrounding lung tissue. This spread to the lung could be easily detected on T1w, T2w as well as LGE images. Scale bars: 5 mm.

Supplemental Figure S3: T_1-, T_2- and T_2*-maps for hearts with teratoma. (A-C) T_1 maps for hearts with a teratoma two month after cell delivery (n=3 rats). Teratomas in the heart had slightly longer relaxation times (801±60 ms) compared to normal myocardium (632±31 ms). **(D-F)** T_2 maps of the same hearts depicted in top row (n=3 rats). The core of teratomas had longer relaxation times (49±8 ms) and was surrounded by a rim with short relaxation times (30±3 ms) compared to normal myocardium (38±2 ms). **(G,I)** While T_2* maps (n=3 rats) showed much lower relaxation times (14±1 ms) at the teratoma rim, relaxation times in the core (36±2 ms) were similar to normal myocardium (35±5 ms). Scale bar: 5 mm.

Supplemental Figure S4: Teratoma growth had no significant effect on cardiac remodeling. (A-E) We did not observe any statistically significant differences between hearts with cell engraftment and teratoma growth (n=5) and hearts without cell engraftment and teratoma growth (Control, n=3). As a general trend, ejection fraction (EF) did increase slightly in both groups with no significant differences between them (P=0.72). **(F)** Scar volumes for hearts with teratoma growth were higher before cell injection, but there was a significant difference in the change of scar volume over time (P=0.05). Control animals showed a small decline in scar volume while teratoma hearts showed an increase in scar volume, particularly from day 28 onwards (teratoma masses were classified as scar). **(G)** Similar to the increases in scar size from

day 28 onwards, left ventricular mass (LVM) was similar between both groups but started to diverge after day 28. This led to a difference in LVM over time, albeit not statistically significant (P=0.06). **(H)** The body weight (BW) for both groups increased over time with similar rates (P=0.48). EDV: end-diastolic volume, ESV: end-systolic volume, SV: stroke volume, CO: cardiac output (Data: mean ± standard deviation).

Supplemental Figure S5: Engraftment of human cardiomyocytes in control hearts. (A) Injection of H7-CMs led to the formation of grafts consisting primarily of human cardiomyocytes (human β-myosin heavy chain: MYHC) at 45 days after cell delivery. **(B)** Human cardiomyocytes started to align themselves to rat cardiomyocytes at the edges of grafts. **(C)** Expression of the gap junction protein Connexin 43 (CON 43) was low indicating the immaturity of these cells. **(D)** Human grafts (human nuclear antigen: HNA) contained few macrophages (CD68), indicating stable engraftment. **(E)** Grafts were vascularized by host-derived blood vessels (CD31). **(F)** Human cardiomyocyte grafts were typically 300-400 µm in thickness and spanned 1-2 mm along the circumference of the rat heart (representative images from one of 4 analyzed rat hearts). **(G)** Protein arrays performed on plasma from an independent cohort of rats (4 animals with confirmed teratoma included) identified enolase-2 and angiopoietin-1 as potential protein based biomarkers. **(H)** Plasma samples from the same animals were used to screen for microRNA based biomarkers. Four microRNAs with statistically significant increases in plasma concentration (*LET-7A, MIR-100, MIR-125, and MIR-126*; P<0.05) were identified. Scale bars: A,B,D,E,F: 100 µm, C: 50 µm.

Supplemental Figure S6: Teratomas were highly proliferative, surrounded by an iron rich rim, and contained cells from all three germ layers. (A,B) H&E images showing two teratomas 56 days after cell transplantation (n=2 representative of five analyzed rat hearts). Most teratomas contained primarily endoderm, some mesoderm and undifferentiated cells with necrosis in the core (n=5 rats). Extensive formation of glands with endodermal appearance was also observed. The teratoma from animal #449 (B) contained primarily undifferentiated cells, some mesoderm and a necrotic core. **(C,D)** Prussian blue (PB) staining confirmed the presence of iron rich cells in the teratoma rim. These cells were likely responsible for the short T_2 and T_2* relaxation times observed on the edges of teratomas. **(E)** A substantial number of human cells (human β1 Integrin: B-INT) were in an active state of the cell cycle (KI67) in line with the proliferative nature of these teratomas. **(F)** Chronic inflammation with macrophages (CD68) along the rim and few macrophages in human cell clusters was observed. **(G)** Teratomas contained cells staining positive for a human specific platelet endothelial cell adhesion molecule marker (CD31). However, these cells did not display endothelial morphology and were likely ESCs. **(H)** Small arteries surrounded by smooth muscle cells (SM22) were abundant in normal myocardium but few were observed in teratomas. SM22 positive, fibroblast-like cells were observed surrounding human cell clusters. We also observed some human cells staining positive for the mesodermal marker SM22. **(I,J)** Teratomas contained human cell clusters (human mitochondria: H-MITO) staining positive for pluripotency markers OCT-3/4 and SOX-2. **(K)** A large number of human cells stained positive for the endodermal transcription factor forkhead box protein A2 (FOXA2). Gland-like structures containing α-fetoprotein (AFP) positive cells (endoderm) were found in most teratomas. **(L)** Small cell clusters staining positive for the ectodermal markers neuron-specific class III beta-tubulin (TUJ-1) and glial fibrillary acidic protein (GFAP) were observed in most teratomas. **(M)** The hypothetical spacing for plasma

collection and MRI is based on the assumption of linear scalability for the detection sensitivity according to differences in plasma volume and imaging resolution between rats and humans. Contamination of a cell product by undifferentiated cells bellow the detection threshold of current assays was assumed, which leads to a long lag time before human detection sensitives are reached. Furthermore, the highest teratoma growth rate reported for humans in the literature was used to estimate teratoma growth. Scale bars: A,B,C,D,E,F,H,I,J,K,L: 1000 µm, G: 100 µm.

SUPPLEMENTAL TABLES

Supplemental Table 1: Cardiac differentiation yields

(n=3)	CTNT positive	CTNT negative
C1-CMs	79 ± 1	21 ± 1
C2-CMs	82 ± 3	18 ± 3
T-CMs	86 ± 2	14 ± 2

Supplemental Table 2: Relaxation parameters

(n=3)	Remote	Teratoma core	Teratoma margin
T_1 [ms]	632 ± 31	801 ± 60	
T_2 [ms]	38 ± 2	49 ± 8	30 ± 3
T_2* [ms]	36 ± 2	35 ± 5	14 ± 1

Supplemental Table 3: MRI measurements for control and teratoma groups

	Day -1		Day 56	
	Control (n=3)	Teratoma (n=5)	Control (n=3)	Teratoma (n=5)
Body weight [g]	236 ± 10	223 ± 19	333 ± 8	321 ± 13
Heart rate [bmp]	384 ± 27	367 ± 27	367 ± 7	384 ± 17
EDV [µl]	319.3 ± 5.3	350.3 ± 50.3	399.4 ± 10.6	447.9 ± 92.5
ESV [µl]	120.1 ± 18.3	171.2 ± 52.4	136.9 ± 23.5	189.0 ± 86.2
SV [µl]	199.2 ± 18.0	179.1 ± 24.7	262.5 ± 13.5	258.9 ± 23.2
EF [%]	62.4 ± 5.7	51.8 ± 9.3	65.8 ± 5.0	59.2 ± 10.4
CO [ml/min]	76.2 ± 1.5	65.4 ± 8.0	96.3 ± 4.1	99.2 ± 7.7
LVM [mg]	411.5 ± 16.8	417.3 ± 39.8	530.8 ± 31.6	614.4 ± 38.7
Scar [µl]	15.7 ± 2.3	39.9 ± 8.4	13.8 ± 7.4	57.0 ± 23.0
Infarct [% of LVM]	4.0 ± 0.7	10.0 ± 1.6	2.7 ± 1.3	9.6 ± 3.4

Supplemental Table 4: MRI measurements for C1-CM, PBS and T-CM treatment groups

	Day -1			Day 28		
	C1-CMs (n=12)	PBS (n=12)	Teratoma (n=10)	C1-CMs (n=12)	PBS (n=12)	Teratoma (n=10)
Body weight [g]	247 ± 15	253 ± 23	222 ± 17	310 ± 17	296 ± 27	261 ± 44
Heart rate [bmp]	406 ± 26	405 ± 22	378 ± 23	375 ± 24	377 ± 29	376 ± 27
EDV [µl]	375 ± 51	396 ± 47	370 ± 45	466 ± 45	497 ± 67	438 ± 77
ESV [µl]	167 ± 33	181 ± 32	179 ± 42	183 ± 33	216 ± 61	194 ± 55
SV [µl]	209 ± 35	216 ± 27	192 ± 30	283 ± 24	281 ± 48	245 ± 45
EF [%]	56 ± 6	55 ± 5	52 ± 8	61 ± 4	57 ± 8	56 ± 8
CO [ml/min]	84 ± 14	87 ± 10	72 ± 12	106 ± 11	106 ± 18	92 ± 15
LVM [mg]	362 ± 28	393 ± 59	385 ± 77	454 ± 65	434 ± 56	456 ± 71
Scar [µl]	45 ± 17	58 ± 18	48 ± 12	46 ± 14	47 ± 17	44 ± 13
Infarct [%LVM]	13 ± 4	15 ± 3	10 ± 2	11 ± 2	11 ± 4	9 ± 2

Supplemental Table 5: List of antibodies used for this study

**Antibody**	**Dilution**	**Vendor**	**Order Nr.**
Donkey secondary antibodies	1:200	Jackson ImmunoResearch	
human cardiac Troponin T (CTNT)	1:200	Abcam	ab45932
human CD31 (PECAM1)	1:100	Dako	M082329-2
human Mitochondria (H-MITO)	1:200	Millipore	MAB1273A4
human Nuclear Antigen (HNA)	1:100	Millipore	MAB1281
human Anti-Integrin β1 (CD29)	1:200	Millipore	MAB1965
human β Myosin heavy chain A4.951	1:100	Dev. Studies hybridoma bank	A4.951
FSP1/S100A4	1:200	Millipore	07-2274
Connexin 43 (CON 43)	1:300	Sigma	C6219
CD31	1:100	Millipore	04-1074
sarcomeric alpha actinin (SAC)	1:100	Sigma	A7811-.2ML
CD68	1:300	Abcam	ab125212
SM22	1:300	Abcam	ab14103
KI67	1:300	Millipore	AB92601
CD90	1:200	Abcam	ab92574
SOX2	1:100	R&D Systems	AF4666
OCT3/4	1:50	BD Biosciences	611203
C-MYC	1:50	Millipore	06-340
NANOG	1:100	eBioscience	14-5768-82
SSEA4	1:200	Abcam	ab16287
β III Tubulin (TUJ-1)	1:100	Abcam	ab14545
HNF-3 β/FOXA2	1:100	R&D Systems	AF2400
α-Fetoprotein (AFP)	1:100	Dako	A0008
Glial fibrillary Acidic Protein (GFAP)	1:100	Dako	Z0334

SUPPLEMENTAL EXPERIMENTAL PROCEDURES

Derivation of human iPSCs

Informed consent was acquired from a patient with dilated cardiomyopathy to collect skin fibroblasts and generate induced pluripotent stem cells (iPSC). Skin fibroblasts were reprogrammed to iPSCs using lentivirus as previously described (Sun et al., 2009). A clone which continued to express reprogramming factors at high levels after differentiating (line T) as well as a clone which did not (line C1) were selected. Alternatively, generation of iPSCs with integration-free Sendai-virus methods (line C2) was performed as previously described (Ebert et al., 2014). Human iPSCs were cultured on Matrigel-coated plates (ES qualified, BD Biosciences, San Diego) using chemically defined E8 medium as previously described (Chen et al., 2011). Cells were passaged using Accutase (Global Cell Solutions) every four days.

Cardiac differentiation

Human iPSCs (lines, T, C1, C2), or H7 ESCs obtained from WiCell (Madison, WI) were grown to 90% confluence. A small molecule-based monolayer technique was employed subsequently for differentiation of human iPSCs or ESCs into beating cardiomyocytes (Ebert et al., 2014; Lian et al., 2012).

Flow cytometry analysis of cardiac markers

CMs were detached as described above and subsequently fixed and permeabilized using BD Cytofix/Cytoperm and BD Perm/Wash kits (BD Biosciences) according to the manufacturer´s instructions. Cells were incubated with a primary mouse anti-cardiac troponin T antibody (CTNT, Thermo Scientific) for 2 hours at 4°C. Afterwards, cells were washed and incubated with a secondary goat anti-mouse IgG (H+L) Alexa 488 antibody (Life Technologies) for 45 min at 4 °C. Cells were washed and analyzed by fluorescent activated cell sorting (FACS; BD Aria II). Data analysis was performed using FlowJo software.

Pluripotency marker analysis

Quantitative real-time PCR (qRT-PCR) for pluripotency markers was performed as described earlier using TaqMan probes (Applied Biosystems / Life Technologies) for *CMYC*, *OCT4*, and *KLF4* (Lan et al., 2013).

Karyotyping

The G-band or GTW banding karyotype at >400 band resolution was performed by the Stanford Cytogenetics Laboratory as described previously (Lan et al., 2013).

Myocardial infarction and cell transplantation

All animal procedures were approved by the ethics committee of Stanford University School of Medicine. Male nude rats (Crl:NIH-Foxn1rnu, n=79) (Charles River Laboratories, Wilmington, MA) aged 8-10 weeks were used for this study. Anesthesia was induced with 3-4% isoflurane in oxygen and was maintained at 1.5-2%. Rats were placed on a heating pad with a feedback controller and maintained at 36±1 °C during the surgical procedure. Pre-emptive analgesia in the form of buprenorphine 0.01-0.05 mg/kg or Carprofen subcutaneous and bupivacaine 0.5-2 mg/kg (local infiltration) was provided as well as eye lubrication ointment. After intubation, rats were ventilated and left thoracotomies were performed between the 3rd and 4th ribs. The pericardial sac was opened and the left anterior descending (LAD) coronary artery was occluded for 1 hour with

a 5-0 Prolene suture close to the left atrial appendage. The chest and the incision were closed after LAD reperfusion. Initial MRI scans were performed 3 days after MI surgery to exclude rats with insufficient infarcts (EF >65%, baseline EF before surgery was 73±1%). All rats fulfilling the inclusion criteria (65/79) were used for cell injections. Four days after MI (one day after initial MRI), a second thoracotomy was performed and $1x10^7$ human CMs or PBSs was injected at three injection sites around the scar area (15 µl per injection site). The following cell and PBS injections were used for different aspects of the study; teratoma detection and growth: T-CMs (n=8), alternative biomarkers: T-CMs (n=8), teratoma detection via LGE: T-CMs (n=16), control for assessment of cardiac function: PBS (n=12), changes in cardiac function due to human CM engraftment: C1-CMs (n=12), and biomarker control group for stable human CM grafts: H7-CMs (n=9). All surgeries were performed by an experienced micro-surgeon (M.W.). Rats received postoperative analgesia for three days and antibiotics via their drinking water (Enrofloxacin, 2.5-4 mg/kg) starting with the first surgery.

***In vivo* magnetic resonance imaging (MRI)**

Imaging was performed 1 day prior and every other week for 2 month after cell delivery, using a preclinical 7T (MR901 Discovery) horizontal bore scanner (Agilent, Santa Clara, CA) with a shielded gradient system (600 mT/m). Rats were anesthetized with isoflurane (3%) and placed onto an animal cradle in prone position. Animals were kept at 37±0.4 °C (during image acquisition) via an air heating system while oxygen and anesthetics (1-2% isoflurane) were supplied via a nose cone (0.5 L/min). Data acquisition was performed with a 4-channel phased array receive only surface coil (Rapid MR International, Columbus, OH) placed around the chest and centered in a decoupled 72 mm transmit/receive volume coil (Agilent). Long- and short-axis scout images were acquired to define the two- and four-chamber long-axis views. The cine long-axis views were used to define the short-axis orientation. A prospectively double gated (ECG and respiration) spoiled gradient echo sequence was used to acquire cine cardiac images with the following parameters for standard cine acquisitions: TE 1.5 ms, TR 6-8 ms, flip angle 15°, slice thickness 1 mm, no slice separation, FOV 50×50 mm^2, matrix size 192×192, NSA 1 for short-axis and 2 for long-axis. Twenty cine-frames were recorded to cover the cardiac cycle. A single short-axis slice was obtained in approximately 45 seconds, leading to a total scan time of 11-13 min covering the heart from base to apex (14-15 slices). A double gated spin-echo sequence was used for T_1-weighted acquisitions using the following imaging parameters: TE 9.7 ms, TR 700 ms, slice thickness 1 mm, no slice separation, FOV 40×40 mm^2, matrix size 192×192, NSA 4. For T_2-weighted acquisitions, a double gated spin echo sequence with the following imaging parameters was used: TE 20 ms, TR 700 ms, slice thickness 1 mm, no slice separation, FOV 40×40 mm^2, matrix size 192×192, NSA 4. A fast spoiled gradient echo sequence with the following imaging parameters was used to acquire T_2*-weighted images: TE 7 ms, TR 10.3 ms, flip angle 20°, slice thickness 1 mm, FOV 40×40 mm^2, matrix size 192×192, NSA 3. After cine, T_1-weighted, T_2-weighted and T_2*-weighted acquisitions, gadolinium was injected via an i.v. infusion line (0.8 mmol/kg, Magnevist Bayer, Germany). Late gadolinium enhancement (LGE) images were acquired 8 min after i.v. infusion using an inversion recovery gradient echo sequence with inversion time optimized to null the signal from healthy myocardium (Price et al., 2011). Imaging parameters for these acquisitions were as follows: TE 1.4 ms, TR one breathing interval, TI 280-370 ms, flip angle 90°, slice thickness 1 mm, no slice separation, FOV 40×40 mm^2, matrix size 192×192, NSA 2, views per segment 2. The acquisition time was roughly 1 min per slice. The imaging protocol for one rat typically required 70 min.

Ex vivo MRI

Rat hearts were fixed in 4% PFA over night at 4 °C. Following fixation, hearts were embedded in 2% low melting point agarose in 50 ml tubes. Imaging was performed using a preclinical 7T horizontal bore scanner with a shielded gradient system and a 30 mm inner diameter Millipede coil (Agilent, Santa Clara, CA). For T_1 mapping, a series of multi-slice spin echo images was acquired using the following imaging parameters: TE 8 ms, TR 4000 ms, TI 50, 100, 200, 300, 400, 800 ms, slice thickness 0.5 mm, 0.5 mm gap, FOV 25×25 mm², matrix size 256×256, NSA 1. For T_2 mapping, a series of multi-slice spin echo images was acquired using the following imaging parameters: TE 10, 15, 20, 30, 40, 76 ms, TR 4000 ms, slice thickness 0.5 mm, 0.5 mm gap, FOV 25×25 mm², matrix size 256×256, NSA 1. For T_2* mapping, a series of spoiled gradient echo images was acquired using the following imaging parameters: TE 5, 10, 15, 18 ms, TR 4000 ms, flip angle 40°, slice thickness 0.5 mm, 0.5 mm gap, FOV 25×25 mm², matrix size 256×256, NSA 1. T_1, T_2 and T_2* maps were generated by fitting the spin echo and gradient echo signal equations using a custom Matlab script. Free hand regions of interest were drawn to estimate average relaxation parameters for remote myocardium, center of teratoma and teratoma rim.

MRI data analysis

For the assessment of cardiac function, cine images from one animal were combined to a dataset, randomized, and anonymized. Data analysis was performed using the semi-automatic segmentation software Segment (Medviso AB, Sweden) (Heiberg et al., 2010) as previously described (Riegler et al., 2010). For the detection of possible teratomas and the quantification of teratoma volume, acquisitions from weeks 2, 4, 6 and 8 were paired with the pre-scan (day -1) for each animal and blinded. Regions of interest (ROI) were drawn manually by an experienced user outlining suspected teratomas using ImageJ. Cine images were scored as teratoma positive or negative without volumetric quantification.

Ultrasound imaging

B-mode ultrasound imaging was performed at the same days as MRI was performed. Rats were anesthetized using 2% isoflurane and imaged in a supine position on a heated platform. Imaging was performed using a real-time microvisualization transducer (MS250) with a frequency of 25 MHz connected to a Vevo 2100 ultrasound system (Visualsonics, Toronto, Canada). Three to four B-mode cine images were acquired in long-axis orientation and four to five in short-axis orientation. Acquired data was scored by a blinded observer for visible signs of teratoma.

Immunodetection and histological methods

Heart fixation and section preparation: Rats were anesthetized, 1 ml of heparin (1000 Units, APP Pharmaceuticals, IL) was injected i.v, the chest was opened, a 25G butterfly needle inserted into the left ventricle via the apex, and 6 ml of blood were collected in EDTA tubes. Following blood collections, cuts into the liver were made and their hearts were perfused with 50 ml cold PBS (4 ºC) containing 0.1 mol/l KCl. Hearts were excised and fixed over-night at 4 ºC in PBS with 4% PFA. Following fixation, hearts were transferred into 30% sucrose solution and kept at 4 ºC until equilibrium was reached. Hearts were embedded in OCT and frozen in hexane containing dry ice. Sections were cut with a cryostat (Leica, Wetzlar, Germany) collected on glass slides, dried and stored at -80 ºC.

Preparation of cell samples: Human iPSCs or iPSC-CMs were seeded in chambered coverslides (Lab-Tek, Nalge Nuc International, NY) and cultured for 3-4 days. Cells were washed once with PBS before fixation with 4% PFA for 10 min at room temperature. After fixation, cells were permeabilized for 15 min with PBS containing 0.1% Triton-X at room temperature. Immunostaining was performed as outlined below.

Immunofluorescence: Sections were equilibrated to room temperature, washed 3x 10 min with PBS, permeabilized with 0.5% Triton-X (Sigma) in PBS for 60 min at room temperature followed by incubation in blocking solution (5% donkey serum in PBS + 0.1% Tween20, Sigma) for 60 min. Sections were incubated with primary antibodies (see Supplemental Table S5), and diluted in blocking solution over night at 4 ºC in a humid chamber. After washing 3x 15 min with PBS + 0.1% Tween20, sections were incubated with secondary antibodies diluted in blocking solution for 1 hour followed by a final washing step (3x 15 min in PBS + 0.1% Tween20) and covered with cover slips using self-hardening mounting media. Confocal microscopy was performed using a Leica SP8 microscope (Leica, Wetzlar, Germany). A series of images was acquired with a 20x or 63x oil immersion objective. Images were stitched together to generate composite images to display the entire cross-section of a teratoma.

Screening for cell engraftment: Hearts were cut from apex to base. All sections were collected and mounted on glass slides. Sections on every third slide, advancing the distance from apex to base by 360 μm, were stained against human integrin β1 and human nuclear antigen. Stained slides were manually screened for human grafts using a fluorescence microscope with a 20x objective (Nikon, Japan).

Quantification of plasma biomarkers

Approximately 2 ml of blood were collected directly after each MRI session via a tail vein catheter using EDTA coated tubes (BD, NJ). The final blood collection was performed after terminal anesthesia as described above. Blood was stored on ice for up to 3 hours before centrifugation at 3000g to separate the plasma. Plasma was aliquoted (100 μl per tube) and stored at -80 °C until specific ELISAs were performed. Plasma samples were diluted 1:1 with the respective sample dilution buffers provided. Two or three wells were used for each sample on each ELISA plate. The following ELISA kits were acquired and performed following manufacturers protocols: carcinoembryonic antigen (CEA, Abcam: ab99992), α-fetoprotein (AFP, R&D Systems: DAFP00), fibroblast growth factor (FGF, Invitrogen: KHG0021), human chorionic gonadotropin (HCG, Sigma: RAB0092), vascular endothelial growth factor (VEGF, Sigma: RAB0508), lactate dehydrogenase (LDH, Abcam: ab116693), alkaline phosphatase (ALP, Abcam: ab83369 and RayBiotech: ELH-ALKP-1), and cancer antigen-125 (CA-125, Abcam: ab108653). Absorbance was measured at the recommended wavelength for the different ELISAs using a plate reader (GloMax-Multi, Promega, WI).

RNA Extraction and cDNA Synthesis

Total RNA, including miRNA, was extracted from 150 µl serum by using a miRNeasy Serum/Plasma Kit (Qiagen) according to the manufacturer's protocol. Briefly, five volumes of QIAzol lysis reagent along with a synthetic spike-in control, *Caenorhabditis elegans* miR-39 (1.6 × 108 copies/µl), was added to the lysed samples for internal normalization. After adding an equal volume of chloroform, the samples were centrifuged for 15 min at 12,000 g at 4°C. The upper aqueous phase was mixed with 1.5 volumes of 100% ethanol, transferred to a spin column, centrifuged, washed, and eluted in 14 µl RNase-free water. 2 µl of each RNA sample was used

for cDNA synthesis using the miScript II RT kit (QIAGEN). The reaction mixture was incubated for 60 min at 37°C, followed by denaturation for 5 min at 95°C. Each cDNA was further diluted to 200 µl with RNase-free water and stored at -20°C until use.

microRNA Screening

Screening was performed using the Human Cancer Pathway Finder miRNA PCR array (Qiagen) according to the manufacturer's protocol. All the miRNAs included in the arrays have previously been reported to be implicated in various forms of cancers. Each array included C. elegans miR-39 primer assays for internal normalization, positive PCR control (PPC) assays, and miRNA reverse transcription control (miRTC) assays. The data sets were calibrated using a C. elegans *miR-39* assay, which detected the spike-in control that was added to the serum samples during RNA extraction. An Excel-based miRNA PCR Array Data Analysis tool (Qiagen; http://pcrdataanalysis.sabiosciences.com/mirna/arrayanalysis.php) was used for data analysis.

Proteome profiler array

Screening was performed using the Human XL Oncology Array Kit array (R&D Systems: ARY026) according to the manufacturer's protocol using 500 µl of plasma for each time point.

SUPPLEMENTAL REFERENCES

Chen, G., Gulbranson, D.R., Hou, Z., Bolin, J.M., Ruotti, V., Probasco, M.D., Smuga-Otto, K., Howden, S.E., Diol, N.R., Propson, N.E., *et al.* (2011). Chemically defined conditions for human iPSC derivation and culture. Nat Methods *8*, 424-429.

Ebert, A.D., Kodo, K., Liang, P., Wu, H., Huber, B.C., Riegler, J., Churko, J., Lee, J., de Almeida, P., Lan, F., *et al.* (2014). Characterization of the molecular mechanisms underlying increased ischemic damage in the aldehyde dehydrogenase 2 genetic polymorphism using a human induced pluripotent stem cell model system. Sci Transl Med *6*, 255ra130.

Heiberg, E., Sjogren, J., Ugander, M., Carlsson, M., Engblom, H., and Arheden, H. (2010). Design and validation of Segment--freely available software for cardiovascular image analysis. BMC Med Imaging *10*, 1.

Lan, F., Lee, A.S., Liang, P., Sanchez-Freire, V., Nguyen, P.K., Wang, L., Han, L., Yen, M., Wang, Y., Sun, N., *et al.* (2013). Abnormal calcium handling properties underlie familial hypertrophic cardiomyopathy pathology in patient-specific induced pluripotent stem cells. Cell Stem Cell *12*, 101-113.

Lian, X., Hsiao, C., Wilson, G., Zhu, K., Hazeltine, L.B., Azarin, S.M., Raval, K.K., Zhang, J., Kamp, T.J., and Palecek, S.P. (2012). Robust cardiomyocyte differentiation from human pluripotent stem cells via temporal modulation of canonical Wnt signaling. Proc Natl Acad Sci U S A *109*, E1848-1857.

Price, A.N., Cheung, K.K., Lim, S.Y., Yellon, D.M., Hausenloy, D.J., and Lythgoe, M.F. (2011). Rapid assessment of myocardial infarct size in rodents using multi-slice inversion recovery late gadolinium enhancement CMR at 9.4T. J Cardiovasc Magn Reson *13*, 44.

Riegler, J., Cheung, K.K., Man, Y.F., Cleary, J.O., Price, A.N., and Lythgoe, M.F. (2010). Comparison of segmentation methods for MRI measurement of cardiac function in rats. J Magn Reson Imaging *32*, 869-877.

Sun, N., Panetta, N.J., Gupta, D.M., Wilson, K.D., Lee, A., Jia, F., Hu, S., Cherry, A.M., Robbins, R.C., Longaker, M.T., *et al.* (2009). Feeder-free derivation of induced pluripotent stem cells from adult human adipose stem cells. Proc Natl Acad Sci U S A *106*, 15720-15725.
