# Supplementary material for: Comparison of Magnetic Resonance Imaging and Serum Biomarkers for Detection of Human Pluripotent Stem Cell-Derived Teratomas
Source: Stem Cell Reports. 2016 Jan 14;6(2):176–87. doi: 10.1016/j.stemcr.2015.12.008 (PMC4750097; doi:10.1016/j.stemcr.2015.12.008)
Supplement: Document S2. Article plus Supplemental Information [file mmc2.pdf]

Stem Cell Reports
Article
OPEN ACCESS

Comparison of Magnetic Resonance Imaging and Serum Biomarkers for Detection of Human Pluripotent Stem Cell-Derived Teratomas

Johannes Riegler,[1,2,5] Antje Ebert,[1,2,5] Xulei Qin,[1,2] Qi Shen,[1] Mouer Wang,[1] Mohamed Ameen,[1] Kazuki Kodo,[1,2] Sang-Ging Ong,[1,2] Won Hee Lee,[1,2] Grace Lee,[1] Evgenios Neofytou,[1,2] Joseph D. Gold,[1] Andrew J. Connolly,[3] and Joseph C. Wu[1,2,4,*]
[1]Stanford Cardiovascular Institute, Stanford University School of Medicine, Lorry Lokey Stem Cell Research Building, 265 Campus Drive, Stanford, CA 94305, USA
[2]Division of Cardiology, Department of Medicine
[3]Department of Pathology
[4]Molecular Imaging Program, Department of Radiology
Stanford University School of Medicine, Stanford, CA 94305, USA
[5]Co-first author
*Correspondence: joewu@stanford.edu
http://dx.doi.org/10.1016/j.stemcr.2015.12.008
This is an open access article under the CC BY license (http://creativecommons.org/licenses/by/4.0/).

SUMMARY

The use of cells derived from pluripotent stem cells (PSCs) for regenerative therapies confers a considerable risk for neoplastic growth and teratoma formation. Preclinical and clinical assessment of such therapies will require suitable monitoring strategies to understand and mitigate these risks. Here we generated human-induced pluripotent stem cells (iPSCs), selected clones that continued to express reprogramming factors after differentiation into cardiomyocytes, and transplanted these cardiomyocytes into immunocompromised rat hearts post-myocardial infarction. We compared magnetic resonance imaging (MRI), cardiac ultrasound, and serum biomarkers for their ability to delineate teratoma formation and growth. MRI enabled the detection of teratomas with a volume >8 mm^3. A combination of three plasma biomarkers (CEA, AFP, and HCG) was able to detect teratomas with a volume >17 mm^3 and with a sensitivity of more than 87%. Based on our findings, a combination of serum biomarkers with MRI screening may offer the highest sensitivity for teratoma detection and tracking.

INTRODUCTION

The loss of cardiomyocytes following myocardial infarction (MI) leads to reduced force generation that may culminate in heart failure and death (Jessup and Brozena, 2003). Without effective treatment options available to restore lost myocytes, emerging regenerative cell therapies have gained interest. Recent advances in our ability to derive and expand pluripotent stem cells (PSCs) as well as to differentiate them to different cell types such as cardiomyocytes have raised hopes that these cells might be able to replace cells lost during injury or disease and restore organ function (Burridge et al., 2012, 2014).

Preclinical and clinical studies have demonstrated that embryonic stem cell-derived cardiomyocytes (ESC-CMs) or induced PSC-derived cardiomyocytes (iPSC-CMs) alone or as cell sheets can engraft in recipient hearts and improve cardiac function (Caspi et al., 2007; Laflamme et al., 2007; Riegler et al., 2015; van Laake et al., 2007). These and other encouraging findings have led to an increased effort to investigate the safety of ESC-CMs and iPSC-CMs in preclinical animal models and in phase I clinical trials (Menasche et al., 2015). The safety of adult stem cell transplantation into the heart (primarily bone marrow cells) has been established, but to date functional improvements have been underwhelming (Fisher et al., 2014).

In contrast to bone marrow cells and other adult stem cell populations with limited growth and differentiation potential, ESCs and iPSCs have unlimited growth and differentiation potential due to their pluripotent nature (Takahashi and Yamanaka, 2006; Thomson et al., 1998). However, transplantation and engraftment of undifferentiated PSCs can lead to the formation of teratomas, which are typically benign neoplastic growths comprised of tissues from all three germ layers (Ben-David and Benvenisty, 2011; Lee et al., 2013). Hence, it is highly unlikely that ESCs or iPSCs would be used clinically without prior differentiation to a specific cell type (e.g., cardiomyocytes, oligodendrocytes, retinal pigment epithelium). Differentiation limits the proliferative potential of PSCs, but contamination of differentiated cells with undifferentiated or partially differentiated cells still poses a major risk factor for inadvertent tumor or teratoma formation. In addition, there is the possibility that cells might acquire mutations during cultivation or de-differentiate following transplantation (Baker et al., 2007; Mayshar et al., 2010). Since several groups around the world are initiating clinical trials using

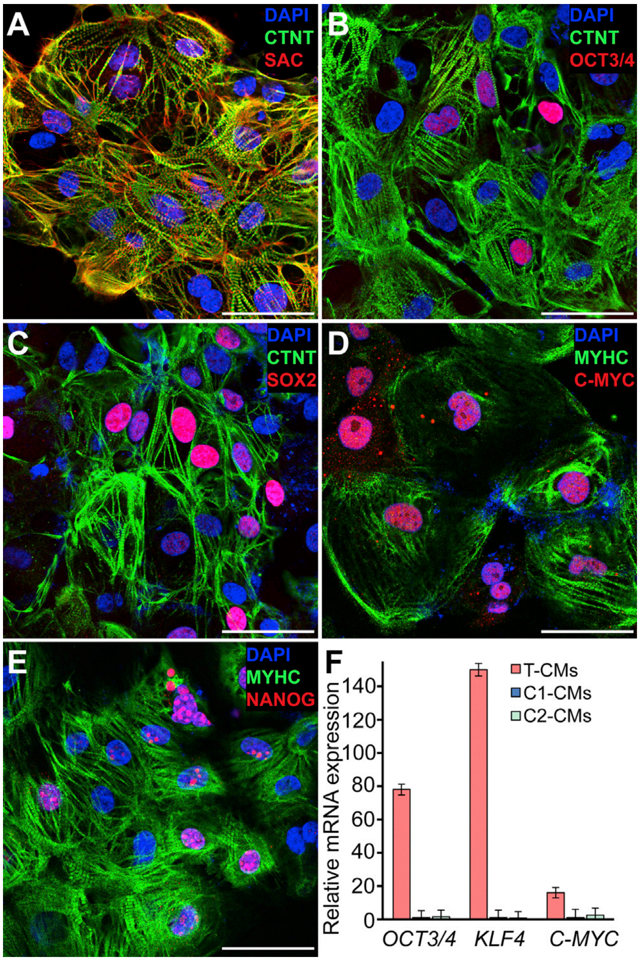

Figure 1. Human iPSC-Derived Cardiomyocytes Showed a Normal Phenotype but Continued to Express Pluripotency Markers

(A) Confocal microscopy image of lentiviral-derived iPSC (clone T)-generated cardiomyocytes (T-CMs) illustrating that differentiated cells consisting primarily of cardiomyocytes with normal sarcomeric structure. CTNT, cardiac troponin T; SAC, sarcomeric alpha actinin.

(B–E) Although cells were clearly expressing cardiac markers (CTNT; β-myosin heavy chain [MYHC]), a substantial number of T-CMs continued to express pluripotency markers OCT3/4, SOX2, and NANOG as well as the cell-cycle gene C-MYC.

(F) Real-time PCR confirmed the overexpression of pluripotency markers in the selected lentiviral-derived T-CMs in contrast to control cardiomyocytes from a lentiviral-derived iPSC clone (C1) or Sendai virus-derived clone (C2) (mean ± SD, n=3 independent differentiations lots for each cell line).

Scale bars represent 50 μm.

PSC-derived cells (Garber, 2013; Neofytou et al., 2015), there is an urgent need to establish suitable monitoring strategies to enable early detection of neoplastic growth or teratoma formation. Ideally, such a monitoring strategy should be non-invasive and should not require genetic modification of the cell product. Since naturally occurring teratomas in humans are rare, little is known about the detection limit of different imaging modalities or serum biomarkers that can aid in the early identification of neoplastic growth or teratoma formation.

To address this bottleneck, we identified a human lentiviral-derived iPSC clone that continued to express reprogramming factors following differentiation, predisposing cells from this clone to revert to a pluripotent state and leading to efficient teratoma formation. We transplanted iPSC-CMs from this line into rat hearts 4 days after MI. In order to detect and track teratoma growth, MRI and ultrasound imaging combined with serial plasma collections were performed every other week for 2 months. We tested eight serum biomarkers (carcinoembryonic antigen, α-fetoprotein, fibroblast growth factor, chorionic gonadotropin, vascular endothelial growth factor, lactate dehydrogenase, alkaline phosphatase, and cancer antigen-125) as well as cancer protein and microRNA arrays for their ability to detect teratomas and analyzed their correlations with teratoma size. Finally, we verified underlying MRI spin-lattice and spin-spin relaxation rates by acquiring parametric maps and characterized teratomas histologically.

RESULTS

Lentiviral-Derived iPSC-CMs Continued to Express Pluripotency Markers

Lentiviral-based gene delivery can lead to random integration of delivered genes, which may cause continued overexpression or reactivation of reprogramming factors. Such cell lines can be tumorigenic in vivo. We generated several lentiviral iPSC clones, differentiated them to cardiomyocytes, and selected a clone that continued to express reprogramming genes due to random integration (clone T) and a control lentiviral clone that did not (clone C1). In addition, we used non-integrating Sendai virus-based reprogramming to generate a second control line (clone C2). None of the cell lines differed in their ability to generate cardiomyocytes with high yields (>80%, n = 3, Table S1). Cardiomyocytes from lentiviral clone T (T-CMs) displayed normal sarcomeric organization, but surprisingly some of the CMs continued to express *OCT3/4*, *SOX2*, and *NANOG*, while almost all CMs were positive for *C-MYC* (Figures 1A–1E). Gross examination for aneuploidies via G-band karyotyping did not reveal any abnormalities (data not shown). Substantial overexpression of pluripotency genes in CMs from line T was confirmed by real-time PCR (Figure 1F). We also stained T-CMs and C2-iPSCs for stage-specific embryonic antigen 4 (SSEA4) that could not be detected on T-CMs (data not shown). Additional images depicting CMs stained for

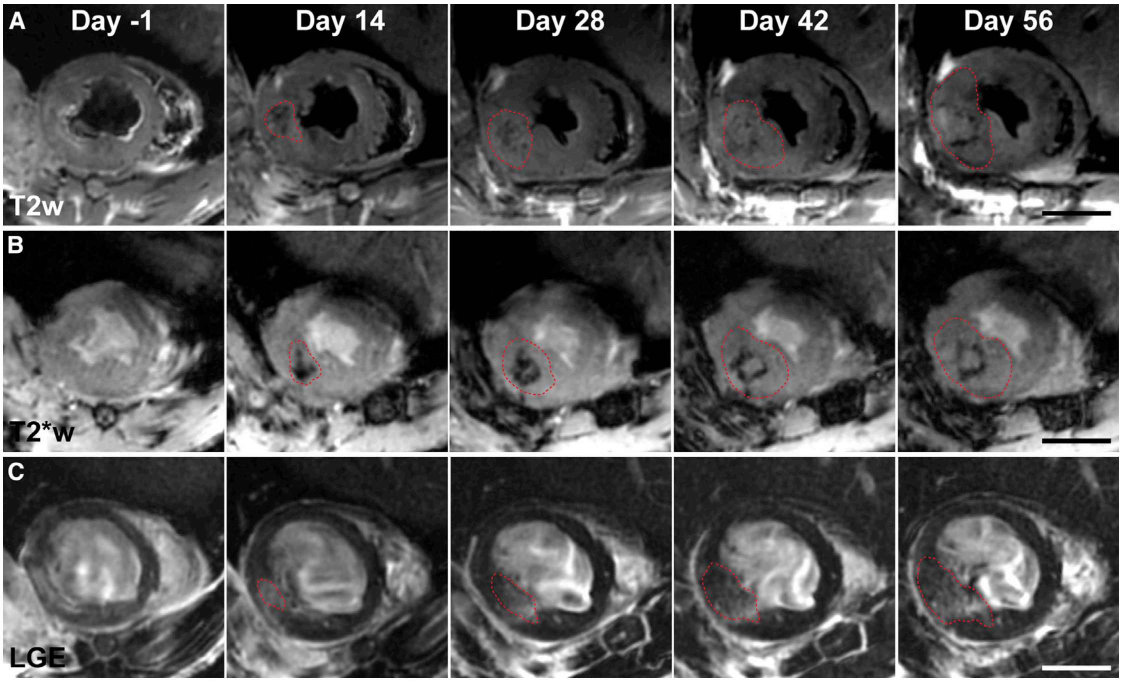

Figure 2. Serial MRI Using T_2w, T_2*w, and LGE Showed the Appearance and Continued Growth of Teratomas

(A) Representative T_2w images of a rat heart 1 day prior and 2, 4, 6, and 8 weeks after cell delivery. A small teratoma with short relaxation times in the core surrounded by a hyperintense rim could be seen from week 2 onward.

(B) T_2*w images for the same teratoma showed a small teratoma 2 weeks after cell injection surrounded by hypointense voxels in the rim. This hypointense rim was gradually pushed outward as the teratoma continued to grow.

(C) LGE images depicted increased left ventricular wall thickness from week 2 onward with hyperintense areas containing non-enhanced clusters illustrating the internal heterogeneity of teratomas.

Dashed red lines outline approximate teratoma boundaries. Scale bars represent 5 mm.

pluripotency markers from both T and C2 lines can be found in Figure S1.

T_2-Weigthed, T_2*-Weighted, and Delayed Enhancement MRI Enabled Teratoma Detection 2 Weeks after Cell Delivery

Little is known about detection limits and the suitability of different imaging sequences for tracking of teratomas that might arise in the heart after cell therapy. To address this, we induced MI in rats by occluding the left anterior descending coronary artery for 1 hr followed by reperfusion. Each animal had a baseline MRI and ultrasound 3 days after MI and received an intramyocardial injection of 1×10^7 T-CMs a day later. Following cell delivery, rats were imaged every 2 weeks for 2 months.

We were able to detect teratomas as soon as 2 weeks after cell delivery on T_2-weighted images (T_2w). Teratomas presented as hyperintense regions with a hypointense rim. Serial imaging showed continued growth of teratomas (Figure 2A). On T_2*-weighted images (T_2*w), teratomas could be primarily discerned by a hypointense core appearing early after cell delivery that persisted as a hypointense rim as the mass continued to grow (Figure 2B). Teratoma appearance on late gadolinium enhancement images (LGE) was slightly more varied. Small masses typically presented as hyperintense regions. However, as masses continued to grow, contrast enhancement declined and small clusters with enhancement rates close to normal myocardium appeared, indicating high cell densities (Figure 2C). Teratomas could be detected only indirectly on T_1-weighted images (T1w) or gradient echo cine images via increased myocardial wall thickness compared with baseline scans (Figures S2A–S2C). In contrast, the low signal to noise ratio of ultrasound images made the detection of masses more difficult. But unusual structures in the myocardial wall or lumen could be detected once teratomas had reached a sufficient size (>50 mm^3; Figure S2D). Imaging at later time points illustrated the ability to detect teratoma spreading to the surrounding lung tissue (Figures S2E and S2F). We next performed T_1, T_2, and T_2* mapping on explanted hearts to verify underlying relaxation rates responsible for teratoma appearance with different contrast weighting (Figure S3). T_2 mapping confirmed long relaxation times for teratoma cores surrounded with

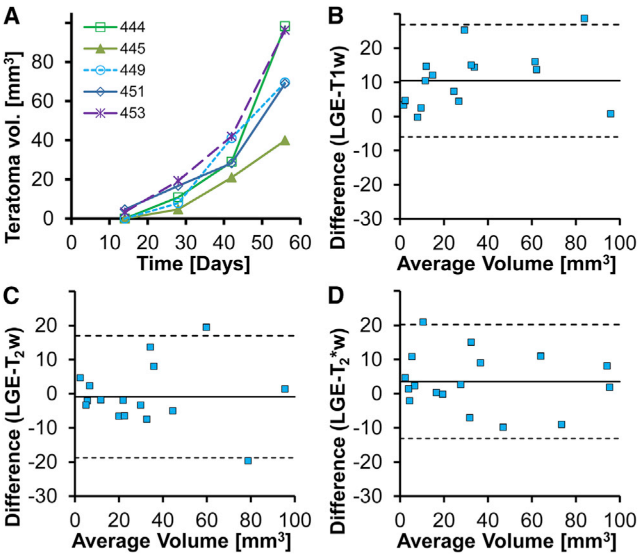

Figure 3. Teratoma Volume Estimates from Different MRI Sequences Were Comparable

(A) Teratoma volume estimates from LGE images showed similar exponential growth for different animals receiving T-CMs (n = 5 rats).

(B) Bland-Altman plot for teratoma volume estimated from LGE and T1w images (n = 5 rats, 4 time points) showed a systematically lower volume estimate for T1w images (10 mm^3) and an SD of 16 mm^3.

(C and D) Bland-Altman plots for T_2w and T_2*w imaging (n = 5 rats, 4 time points) showed little offset between LGE and T_2w or T_2*w volume estimates (−1 and 4 mm^3, respectively). SDs for the differences between LGE and T_2w or T_2*w volume estimates (n = 5 rats, 4 time points) were also similar (18 and 17 mm^3, respectively).

a rim exhibiting short relaxation times compared to normal myocardium (core, 49 ± 8 ms; rim, 30 ± 3 ms; normal, 38 ± 2 ms; n = 3 hearts). Short relaxation times of the teratoma rim were even more pronounced on T_2* maps (rim, 14 ± 1 ms; normal, 35 ± 5 ms; Table S2).

Teratoma Volumes Were Similar for Different Imaging Sequences

Teratoma volumes were measured via manual segmentation by a blinded observer to assess detection limits and limits of agreement for volume estimates based on different imaging sequences. LGE enabled the detection of teratomas >7 mm^3 and showed exponential growth with similar growth rates for all five teratomas in this experiment (Figure 3A). Volume estimates from T1w images were systematically lower (−10 mm^3) compared with LGE (Figure 3B). Teratoma volumes from T_2w and T_2*w imaging had small systematic differences (−1 and +4 mm^3, respectively) and a similar variability when compared against LGE (Figures 3C and 3D). A log-linear plot confirmed exponential growth (R^2 = 0.87) with a 10-day doubling time for teratoma volume (n = 5).

Cardiac Function Was Not Affected by Teratoma Growth or Transplantation of Human Cardiomyocytes

Blinded cine data were analyzed to measure potential changes in cardiac function due to teratoma growth. Five out of eight rats that received T-CMs developed teratomas. Histological analysis of hearts from the three animals without cardiac teratomas showed no significant engraftment of human cells in these hearts (screening sections every 360 μm). We did not observe any statistically significant differences in left ventricular end-diastolic volume (p = 0.27), left ventricular end-systolic volume (p = 0.45), or left ventricular ejection fraction (p = 0.72) between hearts with teratomas and hearts without cell engraftment (Figure S4 and Table S3).

In order to evaluate functional benefits from the transplantation of human iPSC-CMs into ischemic rat hearts, C1-CMs (no integration, n = 12) or PBS (n = 12) were injected into the border zone of the injured myocardium 4 days after ischemia reperfusion. Follow-up cardiac MRI 4 weeks after baseline imaging and cell transplantation did not detect any significant differences in left ventricular end-diastolic volume (p = 0.11), left ventricular end-systolic volume (p = 0.14), or left ventricular ejection fraction (p = 0.68) between the PBS and C1-CM transplantation groups (Table S4).

Serum Biomarkers Were Able to Detect Teratomas 4 Weeks after Cell Delivery

We tested plasma collected at each imaging time point (0, 2, 4, 6, 8 weeks after cell delivery) for biomarkers associated with germ cell tumors or general tumors. We found that a combination of two biomarkers, carcinoembryonic antigen (CEA) and human chorionic gonadotropin (HCG), enabled the detection of 87% (7/8) of teratomas once teratomas had reached a volume >40 mm^3 (Figure 4A). The addition of a third biomarker, α-fetoprotein (AFP), improved the detection limit for AFP-positive teratomas to >17 mm^3. The ability of single biomarkers to detect teratomas were as follows: CEA, 75% (6/8); AFP, 50% (4/8); fibroblast growth factor (FGF), 50% (4/8); HCG, 25% (2/8); and CA-125, 25% (2/8). Although there were slight differences in the sensitivity of different biomarkers, their detection limits were between 20 and 50 mm^3. There was considerable heterogeneity between teratomas with some positive for four biomarkers, while others were only positive for one biomarker. We also observed one teratoma (#449) that was negative for all biomarkers tested, despite reaching a size of 70 mm^3. In contrast, all of these

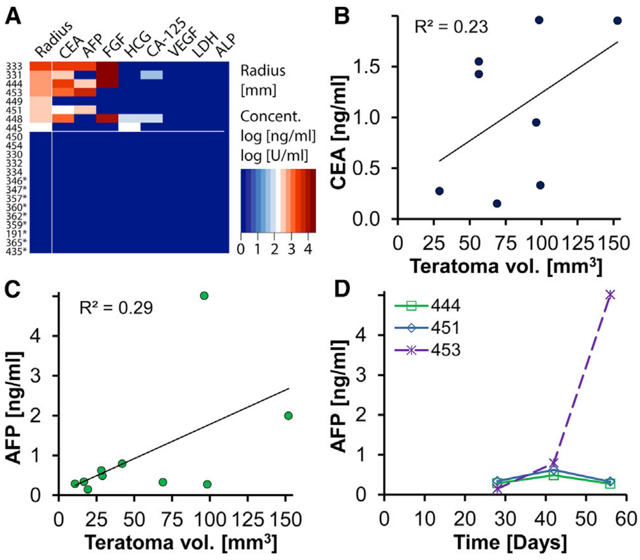

Figure 4. Plasma Biomarkers Were Able to Detect Most Teratomas

(A) Heatmap depicting the volume equivalent radius of eight teratomas 2 months after cell transplantation as well as corresponding plasma levels for a range of biomarkers (n = 8 rats). All of the tested biomarkers were below the detection limit for animals that received either control CMs (H7-derived ESC-CMs, n = 9 rats, asterisk next to animal number) or a subset of T-CMs that had failed to engraft and form a teratoma (n = 5 rats). Teratoma radii are depicted on a linear scale while plasma concentrations are depicted on a log scale.

(B) A weak correlation was found between CEA plasma levels and teratoma volume estimates form LGE MRI ($R^2 = 0.23$, n = 6 rats, 3 time points).

(C) Plasma levels of AFP were also weakly correlated with teratoma volumes ($R^2 = 0.29$, n = 4, 3 time points).

(D) Although AFP could be detected from some teratomas with a volume of less than 20 mm^3, it could not be detected before week 4 following cell delivery (n = 4 rats). Only one of three teratomas for which >3 positive measurements were available showed AFP increases reflecting teratoma growth. VEGF, vascular endothelial growth factor.

biomarkers were below the detection limit for 14 negative control animals. These consisted of five samples from rats where T-CMs failed to engraft (day 30 after cell delivery) and nine samples from rats with confirmed human H7-derived ESC-CM grafts (days 30–190 after cell delivery, Figures S5A–S5F). In addition, we observed loose correlations between CEA or AFP levels and teratoma volumes ($R^2 = 0.23$, $R^2 = 0.29$, Figures 4B and 4C). This was also observed for some animals where biomarker levels increased over time (Figure 4D).

Alternative Serum Biomarkers for Teratoma Detection

An additional set of T-CMs were transplanted into ischemic rat hearts (n = 8) 4 days after ischemia reperfusion injury, followed by plasma collection every other week and MRI at 4 and 8 weeks after cell delivery. Plasma from four animals that developed teratoma was used to screen for cancer-associated microRNAs or proteins. We were able to detect two proteins, enolase-2 and angiopoietin-1, which showed consistent concentration increases over time in three out of four animals with confirmed teratomas (Figure S5G). In addition, we found four microRNAs (*LET-7*, *MIR-100*, *MIR-125*, and *MIR-126*) that showed statistically significant increases 8 weeks after cell transplantation compared to baseline (Figure S5H, $p < 0.05$).

Late Gadolinium Enhancement Acquisitions Were Sufficient for Teratoma Detection

We next set out to assess if LGE alone could be sufficient for teratoma detection in an independent group of animals (n = 16). Blinded analysis of LGE images 1 month after cell delivery identified nine teratomas with 4–57 mm^3 in size. In hearts classified as teratoma free by MRI, histological analysis confirmed the lack of human cell engraftment or teratoma formation. Analysis of hearts with suspected teratomas confirmed all teratomas with volumes >8 mm^3 (Figure 5). However, one heart with a suspected teratoma of 4 mm^3 based on LGE was found to be teratoma free when H&E stainings were analyzed. Taken together, these results indicate that teratomas >8 mm^3 can be detected with LGE based on a voxel size of 0.04 mm^3.

Teratomas Were Poorly Vascularized, Inflamed on the Rim, and Contained Undifferentiated Cells

Immediately after the last imaging session (2 months after cell delivery), hearts were harvested and sectioned for histological examination. H&E staining revealed the presence of undifferentiated cells, endodermal tissue, and some mesodermal tissue as well as areas of necrosis in the core of teratomas (Figures 6A, 6B, S6A, and S6B). We also observed a substantial number of hemosiderin-laden macrophages along the rim of teratomas. Prussian blue staining confirmed that these macrophages contained a substantial amount of iron (Figures 6C, 6D, S6C, and S6D). Closer inspection of teratoma #449, which tested negative for all biomarkers, revealed substantial necrosis within the core as well as the presence of undifferentiated cells with the absence of endodermal or ectodermal tissues and gland-like structures (Figure S6B). KI67 immunofluorescence staining confirmed that the majority of human cells were in an active state of the cell cycle (Figures 6E and S6E), in line with the observed exponential growth rates. Acute and chronic inflammation along the teratoma rim was confirmed by macrophage accumulation. However, only a few macrophages were found inside human cell clusters (Figures 6F and S6F). Although the myocardium is highly vascularized, teratomas contained few blood vessels and

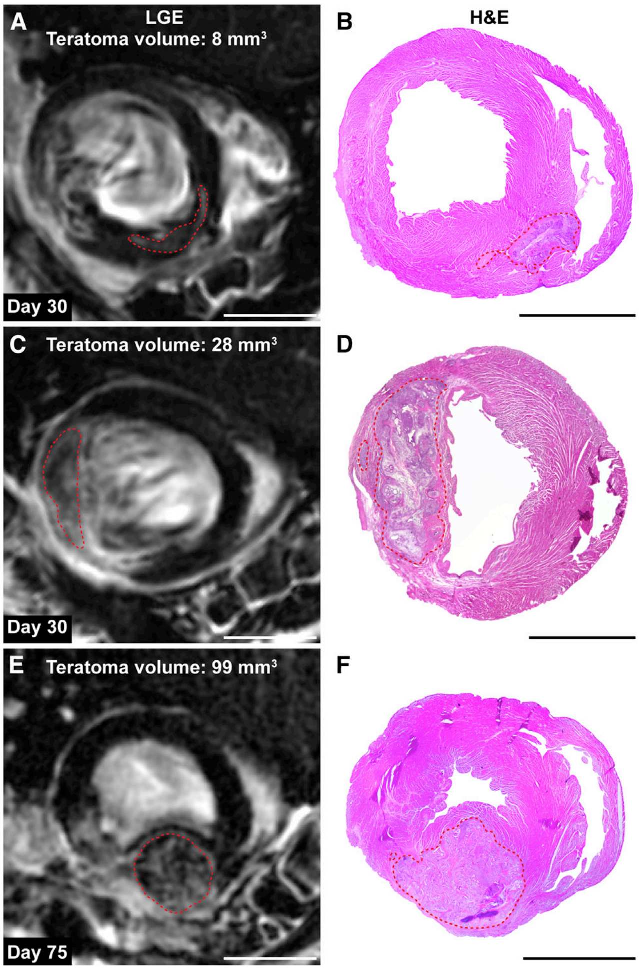

Figure 5. Teratomas Displayed Spatial Heterogeneity on LGE Images

(A–D) LGE images showing a small and a large teratoma 4 weeks after cell delivery. Corresponding H&E images depicting the same teratoma are shown on the right (n = 2 representative hearts of 8). Teratomas are encircled with dashed lines.

(E and F) Teratomas continued to grow and led to spherical displacement of the myocardial wall at day 75 after cell delivery (n = 1 representative heart of 3). Small teratomas contained primarily hyperintense voxels while larger teratomas contained some hyperintense voxels and a large number of voxels that had a T_1 relaxation time close to normal myocardium.

Scale bars represent 5 mm.

only a small number of them were surrounded by smooth muscle cells (Figures 6G, 6H, S6G, and S6H). We also observed human cells staining positive for platelet endothelial cell adhesion molecule (PECAM) marker without showing vascular morphology. These cells are likely to be stem cells since PECAM expression has been reported previously for ESCs (Figure S6G). Immunofluorescence staining confirmed the presence of undifferentiated cells expressing the pluripotency markers OCT3/4 and SOX2 (Figures 6I, 6J, S6I, and S6J). Although T-iPSCs were initially differentiated to cardiomyocytes and mesodermal tissue was found in teratomas, we did not find significant human cardiomyocytes within teratomas (data not shown). A large number of cells staining positive for the endodermal marker forkhead box protein A2 (FOXA2) was found in all teratomas, with some also containing gland-like structures consisting of cells that contained AFP (Figures 6K and S6K). Small clusters of human cells staining positive for the ectodermal markers, neuron-specific class III beta-tubulin (TUJ-1) and glial fibrillary acidic protein (GFAP), were found in most teratomas (Figure 6L). While these teratomas were relatively small and immature, the presence of cells from all three germ layers could be confirmed. In contrast to spontaneously accruing immature human teratomas, our teratomas were lacking neuroepithelial tissues.

DISCUSSION

Pluripotent stem cells offer unprecedented potential for regenerative medicine as they can be differentiated by a specific sequence of signals to become any cell type of the body. At the same time, any cell product generated from them confers a substantial risk for neoplastic growth or teratoma formation (Cunningham et al., 2012). This risk may come from contamination with undifferentiated cells, culture-acquired mutations, or de-differentiation of cells following transplantation (Baker et al., 2007; Lee et al., 2013; Mayshar et al., 2010). While several studies have been undertaken to determine the required number of pluripotent cells in a cell preparation needed to cause teratoma formation (Cao et al., 2007; Lee et al., 2009; Nussbaum et al., 2007), little is known about the sensitivity of potential imaging or serum biomarkers for teratoma detection following cell transplantation.

First-generation iPSC lines were made using lentivirus-based reprogramming due to their efficiency (Takahashi and Yamanaka, 2006). However, the risk for random integration of delivered genes is well known and led to the eventual development of non-integrative reprogramming methods (Aoi et al., 2008; Fusaki et al., 2009). Here we generated a lentiviral-based iPSC line (line T) that continued to express reprogramming factors after differentiation. We reasoned that line T would be an appropriate model for a cell product contaminated with pluripotent cells or for cells with culture-acquired oncogenic transformations. The transcription factors used to induce pluripotency are master regulators of cell states and are accordingly strictly regulated and normally silenced during development (Jaenisch and Young, 2008). Standard safety screening of any cell product derived from pluripotent cells

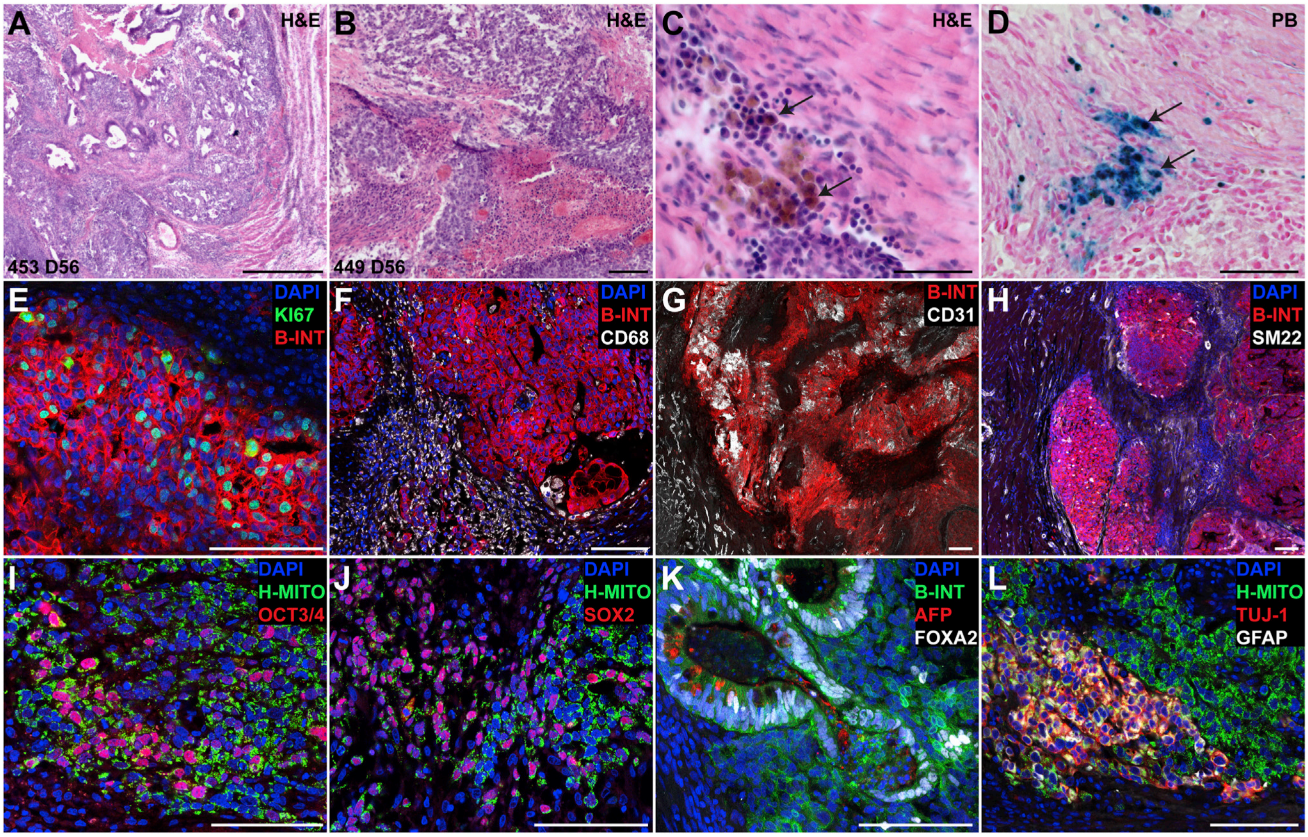

Figure 6. Teratomas Were Highly Proliferative, Inflamed on the Rim, Poorly Vascularized, and Retained Pluripotent Cells

(A and B) H&E images of two teratomas 56 days after cell transplantation. Most teratomas contained primarily endoderm, some mesoderm, and undifferentiated cells with necrosis in the core (n = 5 rats). Extensive formation of glands with endodermal appearance was also observed. The teratoma from animal #449 (B) contained primarily undifferentiated cells, some mesoderm, and a necrotic core.

(C and D) All teratomas were surrounded by hemosiderin-laden macrophages (D, black arrows) along the margin (n = 5 rats). Prussian blue (PB) staining confirmed the presence of iron-rich hemosiderin.

(E) A substantial number of human cells (human β1 integrin [B-INT]) were in an active state of the cell cycle (KI67) in line with the proliferative nature of these teratomas.

(F) Chronic inflammation with macrophage accumulation (CD68) along the rim, but few macrophages infiltrating human cell clusters, was observed.

(G) In contrast to highly vascularized (CD31) myocardial tissue, teratomas were poorly vascularized. Teratomas contained clusters of stem cells staining positive for platelet endothelial cell adhesion molecule (CD31).

(H) Small arteries surrounded by smooth muscle cells (SM22) were abundant in normal myocardium, but few of these were observed in teratomas. SM22 positive, fibroblast-like cells were observed surrounding human cell clusters. We also observed some human cells staining positive for the mesodermal marker SM22.

(I and J) Teratomas contained human cell clusters (human mitochondria [H-MITO]) staining positive for pluripotency markers OCT-3/4 and SOX-2.

(K) A large number of human cells stained positive for the endodermal transcription factor forkhead box protein A2 (FOXA2). Gland-like structures containing AFP-positive cells (endoderm) were found in most teratomas.

(L) Small cell clusters staining positive for the ectodermal markers neuron-specific class III beta-tubulin (TUJ-1) and glial fibrillary acidic protein (GFAP) were observed in most teratomas.

Scale bars represent (A) 500 μm, (B, E–L) 100 μm, (C, D) 50 μm.

will include expression analysis of pluripotency genes; such a screen did easily detect the presence of pluripotent cells in our T-CMs. However, if only a small population expresses these genes or if expression is transiently repressed, detection will become much more difficult. While the risk of aberrant expression of reprogramming factors will be much smaller for iPSC lines generated by non-integrative methods, the risk for contamination of a cell product

with undifferentiated cells or de-differentiation will remain. Current in vitro screening methods can detect down to 1 undifferentiated cell in 100,000 differentiated cells (Tano et al., 2014). Given that regenerative cell therapies for MI may administer up to one billion cells (Chong et al., 2014) and preclinical studies indicate that 1×10^4 to 1×10^5 cells are sufficient to induce teratoma growth (Lee et al., 2009), current procedures for cell purification and characterization cannot ensure that the cell product is free of undifferentiated cells. With limited data on ESC and iPSC-based cell therapies, the clinical risk for teratoma formation is currently unknown. However, the risk for tumor growth from stem cells has been highlighted previously by a patient who developed a brain tumor after a controversial non-US Food and Drug Administration (FDA) approved neuronal stem cell transplantation (Amariglio et al., 2009).

Clinical trials designed to assess the efficacy and safety profile of cardiomyocytes derived from pluripotent cell sources are likely to involve imaging modalities such as MRI or ultrasound to assess cardiac function (Menasche et al., 2015). We therefore decided to assess the suitability of these modalities for safety screening. We were able to detect neoplastic growth (teratoma) reliably using T_2w imaging and LGE once masses had reached a volume of 8 mm^3. Although this sensitivity will decrease with the lower resolution of clinical MRI systems, it is likely to be much lower than average teratoma sizes at the time of detection currently reported (Chang and Lin, 2014; Coleman et al., 2014). Bland-Altman plots showed no systematic offset for teratoma volume estimates based on T_2w imaging or LGE. LGE might be particularly convenient for potential teratoma or neoplastic growth detection since it is frequently included in clinical studies to assess changes in scar size (infarct size). Teratomas were poorly vascularized and surrounded by a hypointense rim on T_2w and T_2*w images. This easy detectable feature was caused by hemorrhage around the teratoma leading to hemosiderin accumulation, which we confirmed histologically. Although short T_2 values have been described previously for human ovarian teratomas, these were primarily due to the formation of bone or fat tissues in mature teratomas (Saba et al., 2009).

Cardiac MRI was performed to assess potential changes in cardiac function due to teratoma growth. For this, a subacute model with cell delivery 4 days after ischemia reperfusion injury was chosen since functional improvements following the transplantation of human ESC-CMs have been reported for this model (Laflamme et al., 2007). T-CM transplantation leading to teratoma growth did not cause statistically significant impairments or improvements in function at our 2-month time point. This might be attributable to still relatively small teratoma sizes (average 76 ± 29 mm^3) at our latest imaging time point. The same animal model was used for a control group that received PBS injections and for C1-CM (no integration of reprogramming factors) transplantations. Follow-up MRI 4 weeks after cell delivery did not detect any functional benefits from C1-CM transplantation in this model. This lack of functional improvements might be due to differences in the animal surgical model, cell preparation, or cell source used.

We were also able to detect teratomas using ultrasound once they had reached a size of 50 mm^3 at 6–8 weeks after cell delivery. The lower sensitivity of ultrasound compared with MRI is consistent with the literature for other organs such as breast (Warner et al., 2001) or liver (Forner et al., 2008), and is likely even more pronounced for the heart where depth penetration and a restricted ultrasound window will further limit accurate detection. Positron emission tomography (PET) or single photon emission computed tomography (SPECT) imaging would offer a higher sensitivity compared to MRI (Nguyen et al., 2014) when specific radiotracers such as arginine-glycine-aspartic acid (RGD) peptide agents, which target angiogenesis, are used (Cao et al., 2009). Another advantage of PET and SPECT is the ability to cover the entire body during an imaging session, whereas MRI typically covers a smaller region such as the head or chest. However, a potential disadvantage is the repeated radiation exposure involving both PET and SPECT radiotracers.

Serum or plasma biomarkers would be preferable over imaging biomarkers because they can be measured more frequently, are more cost effective, and integrate information from the whole body. We chose to assess a range of biomarkers that have been previously associated with germ cell tumors (Polanski and Anderson, 2007). A combination of three biomarkers (CEA, AFP, and HCG) was able to detect 87% (7/8) of teratomas with volumes >17 mm^3. CEA and AFP are both FDA approved as tumor-associated antigens. CEA is a re-expressed oncofetal protein with a sensitivity and specificity of 36% and 87%, respectively, for colorectal cancer (Fletcher, 1986). It is currently used to detect recurrent colorectal cancer and liver metastasis. AFP is an oncofetal protein found in hepatocellular cancer, cirrhosis, and hepatitis. Its sensitivity and specificity for hepatocellular carcinoma are 50% and 70%, respectively (De Masi et al., 2005). HCG is hormone produced during pregnancy and has also been found in choriocarcinoma, testicular cancer, and germ cell tumors. Elevated serum levels are found in 30% of patients with seminoma (a form of testicular cancer), 23% with renal cancer, and 10% with prostate cancer among other types of cancer (Stenman et al., 2004). FGF, which was able to detect 50% of teratomas in our study, is a growth factor with mitogenic, angiogenic, and neutrotrophic properties. FGF is frequently elevated in

melanoma, glioma, and some other cancers. It is considered to be an important factor for tumor angiogenesis and is therefore associated with tumor growth and malignancy (Takahashi et al., 1992). Tumor detection sensitivities of individual serum biomarkers are typically low. Combining several biomarkers can improve the sensitivity to levels suitable for screening. This will be important for teratomas that contain different amounts of tissues from endoderm, mesoderm, or ectoderm, secreting distinct proteins. Finally, we performed exploratory studies for additional novel biomarkers that might be suitable for teratoma detection. We found four microRNAs (*LET-7*, *MIR-100*, *MIR-125*, and *MIR-126*) that could detect teratomas and had previously been associated with oncogenic transformations (Gu et al., 2015; Wu et al., 2015). Furthermore, two proteins (enolase-2 and angiopoietin-1) that had been previously associated with cancer and angiogenesis (Song et al., 2014; Yu et al., 2001) showed increased plasma concentrations that correlated with teratoma growth. Additional research will be required to verify the detection sensitivity of these potential biomarkers. While more sensitive biomarkers would be desirable, they would require some time to be developed for clinical applications and to be approved by US and European regulatory agencies.

Small or immature teratomas are particularly hard to detect due to the lack of tissues with high secretion levels such as gland-like structures. In line with that, we found one teratoma in our test set for which none of the eight tested biomarkers was positive. Histological characterization showed that this particular teratoma had substantial necrosis in the core, did not contain any gland-like structures, and consisted primarily of undifferentiated cells. Immature teratoma will therefore limit the sensitivity of serum biomarkers for early teratoma detection unless a new sensitive biomarker for undifferentiated cells can be found (Ahrlund-Richter and Hendrix, 2014). However, they are likely to be detectable by imaging as this immature teratoma was readily detectable via MRI. Furthermore, screening strategies for regenerative cell therapies should also be able to detect neoplastic growth that might be difficult to detect with serum biomarkers depending on the cell types that are growing. A combination of serum biomarkers and structural imaging should offer a high probability to detect neoplastic growth, as well as immature and mature teratomas. Although detection limits from small-animal studies are difficult to extrapolate to human scale, the larger plasma volume and lower imaging resolution of clinical MRI systems will decrease the sensitivity to detect teratoma. Assuming a similar growth rate for teratomas in humans and a linear decrease in detection sensitivity corresponding to increased plasma volume and decreased image resolution, blood sampling and imaging frequencies could be reduced for humans (Figure S6M). We observed a growth rate of 10 days for the doubling of teratoma volume, which is similar to high growth rates of 11–12 days that have been observed for some human teratomas (Selby et al., 1979). Even with such a high growth rate, it would take several months for a teratoma to reach detection limits in humans as the number of undifferentiated or de-differentiated cells transplanted is likely to be very small. These plasma collection and imaging frequencies would be similar to the sampling strategies employed in clinical trials to assess functional changes following cardiovascular interventions, which should simplify the adaption of such a monitoring strategy to detect neoplastic growth or teratomas.

In summary, regenerative therapies based on PSC-derived cells are rapidly evolving and may finally fulfill their potential and lead to true tissue regeneration. Nonetheless, the residual risk for neoplastic growth or teratoma formation requires a sensitive monitoring strategy. Serum biomarkers can detect most teratomas regardless of the organ in which they reside, but immature teratomas might be missed. To maximize the likelihood for detecting neoplastic growth and teratoma formation, our study suggests that a combination of MRI focusing on high-risk organs with serum biomarkers covering the entire body would be complementary.

EXPERIMENTAL PROCEDURES

An expanded Experimental Procedures section is available in the online Supplemental Information.

Generation of Human iPSCs and Differentiation to Cardiomyocytes

Human iPSCs were generated using lentivirus- or Sendai virus-based reprogramming vectors and were differentiated to cardiomyocytes (see Supplemental Experimental Procedures).

Cryopreservation of CMs and Preparation for Injection

Human ESC-CMs or iPSC-CMs were washed with PBS and detached using TripleE (Gibco). Cells were pelleted at 200 × *g* for 4 min, subsequently re-suspended in fetal bovine serum supplemented with 10% DMSO, and stored for 24 hr at −80°C, before transfer to liquid N_2 for long-term storage. For injections, 1×10^7 viable CMs per animal were thawed briefly at 37°C, re-suspended in culture medium, and pelleted. Cell pellets were re-suspended in 45 μl of PBS and transferred into 0.5-ml insulin syringes with 28G needles.

Ischemia Reperfusion Injury and Cell Transplantation

All experimental protocols were approved by the Stanford Research Ethics Committee. Ischemia reperfusion injury was induced in 8- to 10-week-old male nude athymic rats (n = 65; Charles River) by occluding the left anterior descending coronary artery for 1 hr followed by reperfusion. Surgery was performed aseptically under 1.5%–2% inhaled isoflurane anesthesia. Three

days later, MRI, ultrasound imaging, and plasma collection were performed. One day after baseline imaging (day 4 after MI), a second thoracotomy was performed and 1×10^7 human CMs or PBS was injected at three sites around the scar area (15 μl per injection site). The following cell and PBS injections were used for different aspects of the study: (1) detection and teratoma growth, T-CMs (n = 8); (2) alternative biomarkers, T-CMs (n = 8); (3) teratoma detection via LGE, T-CMs (n = 16); (4) control for assessment of cardiac function, PBS (n = 12); (5) changes in cardiac function due to human CM engraftment, C1-CMs (n = 12); and (6) biomarker control group for stable human CM grafts, H7-CMs (n = 9).

Immunohistochemistry and Histological Methods

Immunofluorescence and histological analyses were performed using standard protocols.

In Vivo MRI

Cine, T1w, T_2w, T_2*w, and LGE images were acquired 1 day before and 2, 4, 6, and 8 weeks after cell delivery using a 7-T MR901 Discovery horizontal bore scanner (Agilent Technologies). The following imaging parameters were used for cardiac cine: fast spoiled gradient echo, echo time (TE) 1.5 ms, repetition time (TR) 6–8 ms, flip angle 15°, slice thickness 1 mm, field of view (FOV) 50×50 mm^2, 20 images per R-R interval, matrix size 192×192, and number of signal averages (NSA) 1. For the remaining acquisitions, a slice thickness of 1 mm, FOV 40×40 mm^2 and a matrix size of 192×192 were used with the following imaging parameters: (1) for T1w acquisitions: spin echo, TE 9.7 ms, TR 700 ms, and NSA 4; (2) for T_2w acquisitions: spin echo, TE 20 ms, TR 700 ms, and NSA 4; (3) for T_2*w acquisitions: spoiled gradient echo, TE 7 ms, TR 10.3 ms, flip angle 20°, and NSA 3; (4) and for LGE: gradient echo inversion recovery, TE 1.4 ms, TR one breathing interval, inversion time 280–370 ms, flip angle 90°, and NSA 2.

Ex Vivo MRI

Fixed hearts were embedded in 2% low melting point agarose and a series of images with different inversion times, T_2-weighting, and T_2*-weighting were acquired using a 30-mm diameter Millipede volume coil (Agilent).

Ultrasound Imaging

B-mode ultrasound imaging was also performed using a Vevo 2100 ultrasound system (Visualsonics).

Quantification of Plasma Biomarkers

Two milliliters of blood were collected directly after each MRI session via a tail vein catheter using EDTA-coated tubes. Blood was stored on ice for up to 3 hr before centrifugation at $3000 \times g$ to separate the plasma. Plasma was aliquoted and stored at −80°C until specific ELISAs were performed. The following ELISA kits were acquired and performed following the manufacturer's protocols: CEA (Abcam), AFP (R&D Systems), FGF (Invitrogen), HCG (Sigma), vascular endothelial growth factor (Sigma), lactate dehydrogenase (LDH; Abcam), alkaline phosphatase (ALP; Abcam), and CA-125 (Abcam). For the exploratory biomarker study, plasma from a separate cohort of animals was used to screen for microRNAs or cancer-associated proteins. Total RNA, including miRNA, was extracted from plasma using an miRNeasy Serum/Plasma Kit (Qiagen) according to the manufacturer's protocol. Screening was performed using the Human Cancer Pathway Finder miRNA PCR array (Qiagen) according to the manufacturer's protocol. The human XL oncology array kit (R&D Systems) was used according to the manufacturer's protocol to screen for potential protein-based teratoma biomarkers.

Statistical Analysis

Results are shown as means ± SD. A regression analysis was performed to test if a linear relation between the natural logarithm of teratoma volume and time exists. A linear mixed effects model was used to test for differences in cardiac function with fixed effects for functional parameter, cell group (teratoma or no cell engraftment), time, and a random effect for individual rats. A regression analysis was performed to test if a linear relationship between plasma biomarker and teratoma volume exists. Four parameter logistic curve fits were performed for absorbance measured from standard dilutions to establish a standard curve and calculate sample concentrations. Bland-Altman plots were generated to compare teratoma volume estimates from different MRI imaging sequences. Statistical analysis was performed using R software version 2.8.1.

SUPPLEMENTAL INFORMATION

Supplemental Information includes Supplemental Experimental Procedures, six figures, and five tables and can be found with this article online at http://dx.doi.org/10.1016/j.stemcr.2015.12.008.

AUTHOR CONTRIBUTIONS

J.R. conceived the study, designed the experiments, implemented the imaging protocols, performed ELISA, analyzed the MRI data, and wrote the manuscript. A.E. generated the iPSC lines, differentiated the cells, characterized the cell lines, and wrote the manuscript. X.Q. and Q.S. contributed to MRI acquisition. M.W. performed the animal surgeries. M.A.A., K.K., and G.L. contributed to cell line generation and characterization. J.R., E.N., S.G.O., and W.H.L. performed the histology and biomarker studies. A.J.C. analyzed and characterized the histological findings. J.D.G. contributed to the experimental design, critical discussions of the experiments, and manuscript writing. J.C.W. conceived the idea and provided experimental advice, manuscript writing, and funding support. All authors reviewed the manuscript.

ACKNOWLEDGMENTS

We would like to thank Laura J. Pisani from the Stanford Small Animal Imaging Facility and Kitty Lee from the Cell Science Imaging Facility for their help with MRI and confocal imaging. This study was supported by an Erwin Schrödinger fellowship from the Austrian Science Fund (FWF) J3314-B23 (J.R.); American Heart Association 11IRG5450017, 12POST12050363 and 14GRNT18970018 (E.N.); American Heart Association 13EIA14420025, National Institutes of Health (NIH) T32 EB009035, HL113006, and California Institute of Regenerative Medicine (CIRM) TR3-05556, RT3-07798, and DR2-05394 (J.C.W.).

Received: May 7, 2015
Revised: December 4, 2015
Accepted: December 7, 2015
Published: January 14, 2016

REFERENCES

Ahrlund-Richter, L., and Hendrix, M.J. (2014). Oncofetal signaling as a target for cancer therapy. Semin. Cancer Biol. *29*, 1–2.

Amariglio, N., Hirshberg, A., Scheithauer, B.W., Cohen, Y., Loewenthal, R., Trakhtenbrot, L., Paz, N., Koren-Michowitz, M., Waldman, D., Leider-Trejo, L., et al. (2009). Donor-derived brain tumor following neural stem cell transplantation in an ataxia telangiectasia patient. PLoS Med. *6*, e1000029.

Aoi, T., Yae, K., Nakagawa, M., Ichisaka, T., Okita, K., Takahashi, K., Chiba, T., and Yamanaka, S. (2008). Generation of pluripotent stem cells from adult mouse liver and stomach cells. Science *321*, 699–702.

Baker, D.E., Harrison, N.J., Maltby, E., Smith, K., Moore, H.D., Shaw, P.J., Heath, P.R., Holden, H., and Andrews, P.W. (2007). Adaptation to culture of human embryonic stem cells and oncogenesis in vivo. Nat. Biotechnol. *25*, 207–215.

Ben-David, U., and Benvenisty, N. (2011). The tumorigenicity of human embryonic and induced pluripotent stem cells. Nat. Rev. Cancer *11*, 268–277.

Burridge, P.W., Keller, G., Gold, J.D., and Wu, J.C. (2012). Production of de novo cardiomyocytes: human pluripotent stem cell differentiation and direct reprogramming. Cell Stem Cell *10*, 16–28.

Burridge, P.W., Matsa, E., Shukla, P., Lin, Z.C., Churko, J.M., Ebert, A.D., Lan, F., Diecke, S., Huber, B., Mordwinkin, N.M., et al. (2014). Chemically defined generation of human cardiomyocytes. Nat. Methods *11*, 855–860.

Cao, F., van der Bogt, K.E., Sadrzadeh, A., Xie, X., Sheikh, A.Y., Wang, H., Connolly, A.J., Robbins, R.C., and Wu, J.C. (2007). Spatial and temporal kinetics of teratoma formation from murine embryonic stem cell transplantation. Stem Cells Dev. *16*, 883–891.

Cao, F., Li, Z., Lee, A., Liu, Z., Chen, K., Wang, H., Cai, W., Chen, X., and Wu, J.C. (2009). Noninvasive de novo imaging of human embryonic stem cell-derived teratoma formation. Cancer Res. *69*, 2709–2713.

Caspi, O., Huber, I., Kehat, I., Habib, M., Arbel, G., Gepstein, A., Yankelson, L., Aronson, D., Beyar, R., and Gepstein, L. (2007). Transplantation of human embryonic stem cell-derived cardiomyocytes improves myocardial performance in infarcted rat hearts. J. Am. Coll. Cardiol. *50*, 1884–1893.

Chang, C.F., and Lin, C.K. (2014). A case of recurrent, bilateral ovarian mature teratoma in a young woman. BMC Womens Health *14*, 57.

Chong, J.J., Yang, X., Don, C.W., Minami, E., Liu, Y.W., Weyers, J.J., Mahoney, W.M., Van Biber, B., Cook, S.M., Palpant, N.J., et al. (2014). Human embryonic-stem-cell-derived cardiomyocytes regenerate non-human primate hearts. Nature *510*, 273–277.

Coleman, A., Shaaban, A., Keswani, S., and Lim, F.Y. (2014). Sacrococcygeal teratoma growth rate predicts adverse outcomes. J. Pediatr. Surg. *49*, 985–989.

Cunningham, J.J., Ulbright, T.M., Pera, M.F., and Looijenga, L.H. (2012). Lessons from human teratomas to guide development of safe stem cell therapies. Nat. Biotechnol. *30*, 849–857.

De Masi, S., Tosti, M.E., and Mele, A. (2005). Screening for hepatocellular carcinoma. Dig. Liver Dis. *37*, 260–268.

Fisher, S.A., Brunskill, S.J., Doree, C., Mathur, A., Taggart, D.P., and Martin-Rendon, E. (2014). Stem cell therapy for chronic ischaemic heart disease and congestive heart failure. Cochrane Database Syst. Rev., CD007888.

Fletcher, R.H. (1986). Carcinoembryonic antigen. Ann. Intern. Med. *104*, 66–73.

Forner, A., Vilana, R., Ayuso, C., Bianchi, L., Sole, M., Ayuso, J.R., Boix, L., Sala, M., Varela, M., Llovet, J.M., et al. (2008). Diagnosis of hepatic nodules 20 mm or smaller in cirrhosis: prospective validation of the noninvasive diagnostic criteria for hepatocellular carcinoma. Hepatology *47*, 97–104.

Fusaki, N., Ban, H., Nishiyama, A., Saeki, K., and Hasegawa, M. (2009). Efficient induction of transgene-free human pluripotent stem cells using a vector based on Sendai virus, an RNA virus that does not integrate into the host genome. Proc. Jpn. Acad. Ser. B Phys. Biol. Sci. *85*, 348–362.

Garber, K. (2013). Inducing translation. Nat. Biotechnol. *31*, 483–486.

Gu, L., Li, H., Chen, L., Ma, X., Gao, Y., Li, X., Zhang, Y., Fan, Y., and Zhang, X. (2015). MicroRNAs as prognostic molecular signatures in renal cell carcinoma: a systematic review and meta-analysis. Oncotarget *6*, 32545–32560.

Jaenisch, R., and Young, R. (2008). Stem cells, the molecular circuitry of pluripotency and nuclear reprogramming. Cell *132*, 567–582.

Jessup, M., and Brozena, S. (2003). Heart failure. N. Engl. J. Med. *348*, 2007–2018.

Laflamme, M.A., Chen, K.Y., Naumova, A.V., Muskheli, V., Fugate, J.A., Dupras, S.K., Reinecke, H., Xu, C., Hassanipour, M., Police, S., et al. (2007). Cardiomyocytes derived from human embryonic stem cells in pro-survival factors enhance function of infarcted rat hearts. Nat. Biotechnol. *25*, 1015–1024.

Lee, A.S., Tang, C., Cao, F., Xie, X., van der Bogt, K., Hwang, A., Connolly, A.J., Robbins, R.C., and Wu, J.C. (2009). Effects of cell number on teratoma formation by human embryonic stem cells. Cell Cycle *8*, 2608–2612.

Lee, A.S., Tang, C., Rao, M.S., Weissman, I.L., and Wu, J.C. (2013). Tumorigenicity as a clinical hurdle for pluripotent stem cell therapies. Nat. Med. *19*, 998–1004.

Mayshar, Y., Ben-David, U., Lavon, N., Biancotti, J.C., Yakir, B., Clark, A.T., Plath, K., Lowry, W.E., and Benvenisty, N. (2010). Identification and classification of chromosomal aberrations in human induced pluripotent stem cells. Cell Stem Cell *7*, 521–531.

Menasche, P., Vanneaux, V., Hagege, A., Bel, A., Cholley, B., Cacciapuoti, I., Parouchev, A., Benhamouda, N., Tachdjian, G., Tosca, L., et al. (2015). Human embryonic stem cell-derived cardiac progenitors for severe heart failure treatment: first clinical case report. Eur. Heart J. *36*, 2011–2017.

Neofytou, E., O'Brien, C.G., Couture, L.A., and Wu, J.C. (2015). Hurdles to clinical translation of human induced pluripotent stem cells. J. Clin. Invest. *125*, 2551–2557.

Nguyen, P.K., Riegler, J., and Wu, J.C. (2014). Stem cell imaging: from bench to bedside. Cell Stem Cell *14*, 431–444.

Nussbaum, J., Minami, E., Laflamme, M.A., Virag, J.A., Ware, C.B., Masino, A., Muskheli, V., Pabon, L., Reinecke, H., and Murry, C.E. (2007). Transplantation of undifferentiated murine embryonic stem cells in the heart: teratoma formation and immune response. FASEB J. *21*, 1345–1357.

Polanski, M., and Anderson, N.L. (2007). A list of candidate cancer biomarkers for targeted proteomics. Biomark Insights *1*, 1–48.

Riegler, J., Tiburcy, M., Ebert, A., Tzatzalos, E., Raaz, U., Abilez, O.J., Shen, Q., Kooreman, N.G., Neofytou, E., Chen, V.C., et al. (2015). Human engineered heart muscles engraft and survive long term in a rodent myocardial infarction model. Circ. Res. *117*, 720–730.

Saba, L., Guerriero, S., Sulcis, R., Virgilio, B., Melis, G., and Mallarini, G. (2009). Mature and immature ovarian teratomas: CT, US and MR imaging characteristics. Eur. J. Radiol. *72*, 454–463.

Selby, P.J., Heyderman, E., Gibbs, J., and Peckham, M.J. (1979). A human testicular teratoma serially transplanted in immune-deprived mice. Br. J. Cancer *39*, 578–583.

Song, Y., Luo, Q., Long, H., Hu, Z., Que, T., Zhang, X., Li, Z., Wang, G., Yi, L., Liu, Z., et al. (2014). Alpha-enolase as a potential cancer prognostic marker promotes cell growth, migration, and invasion in glioma. Mol. Cancer *13*, 65.

Stenman, U.H., Alfthan, H., and Hotakainen, K. (2004). Human chorionic gonadotropin in cancer. Clin. Biochem. *37*, 549–561.

Takahashi, K., and Yamanaka, S. (2006). Induction of pluripotent stem cells from mouse embryonic and adult fibroblast cultures by defined factors. Cell *126*, 663–676.

Takahashi, J.A., Fukumoto, M., Igarashi, K., Oda, Y., Kikuchi, H., and Hatanaka, M. (1992). Correlation of basic fibroblast growth factor expression levels with the degree of malignancy and vascularity in human gliomas. J. Neurosurg. *76*, 792–798.

Tano, K., Yasuda, S., Kuroda, T., Saito, H., Umezawa, A., and Sato, Y. (2014). A novel in vitro method for detecting undifferentiated human pluripotent stem cells as impurities in cell therapy products using a highly efficient culture system. PLoS One *9*, e110496.

Thomson, J.A., Itskovitz-Eldor, J., Shapiro, S.S., Waknitz, M.A., Swiergiel, J.J., Marshall, V.S., and Jones, J.M. (1998). Embryonic stem cell lines derived from human blastocysts. Science *282*, 1145–1147.

van Laake, L.W., Passier, R., Monshouwer-Kloots, J., Verkleij, A.J., Lips, D.J., Freund, C., den Ouden, K., Ward-van Oostwaard, D., Korving, J., Tertoolen, L.G., et al. (2007). Human embryonic stem cell-derived cardiomyocytes survive and mature in the mouse heart and transiently improve function after myocardial infarction. Stem Cell Res. *1*, 9–24.

Warner, E., Plewes, D.B., Shumak, R.S., Catzavelos, G.C., Di Prospero, L.S., Yaffe, M.J., Goel, V., Ramsay, E., Chart, P.L., Cole, D.E., et al. (2001). Comparison of breast magnetic resonance imaging, mammography, and ultrasound for surveillance of women at high risk for hereditary breast cancer. J. Clin. Oncol. *19*, 3524–3531.

Wu, L., Nguyen, L.H., Zhou, K., de Soysa, T.Y., Li, L., Miller, J.B., Tian, J., Locker, J., Zhang, S., Shinoda, G., et al. (2015). Precise expression levels balance organ regeneration against tumor suppression. Elife *4*. http://dx.doi.org/10.7554/eLife.09431.

Yu, Y., Varughese, J., Brown, L.F., Mulliken, J.B., and Bischoff, J. (2001). Increased Tie2 expression, enhanced response to angiopoietin-1, and dysregulated angiopoietin-2 expression in hemangioma-derived endothelial cells. Am. J. Pathol. *159*, 2271–2280.

Stem Cell Reports, Volume *6*

Supplemental Information

Comparison of Magnetic Resonance Imaging and Serum Biomarkers for Detection of Human Pluripotent Stem Cell-Derived Teratomas

Johannes Riegler, Antje Ebert, Xulei Qin, Qi Shen, Mouer Wang, Mohamed Ameen, Kazuki Kodo, Sang-Ging Ong, Won Hee Lee, Grace Lee, Evgenios Neofytou, Joseph D. Gold, Andrew J. Connolly, and Joseph C. Wu

SUPPLEMENTAL FIGURES

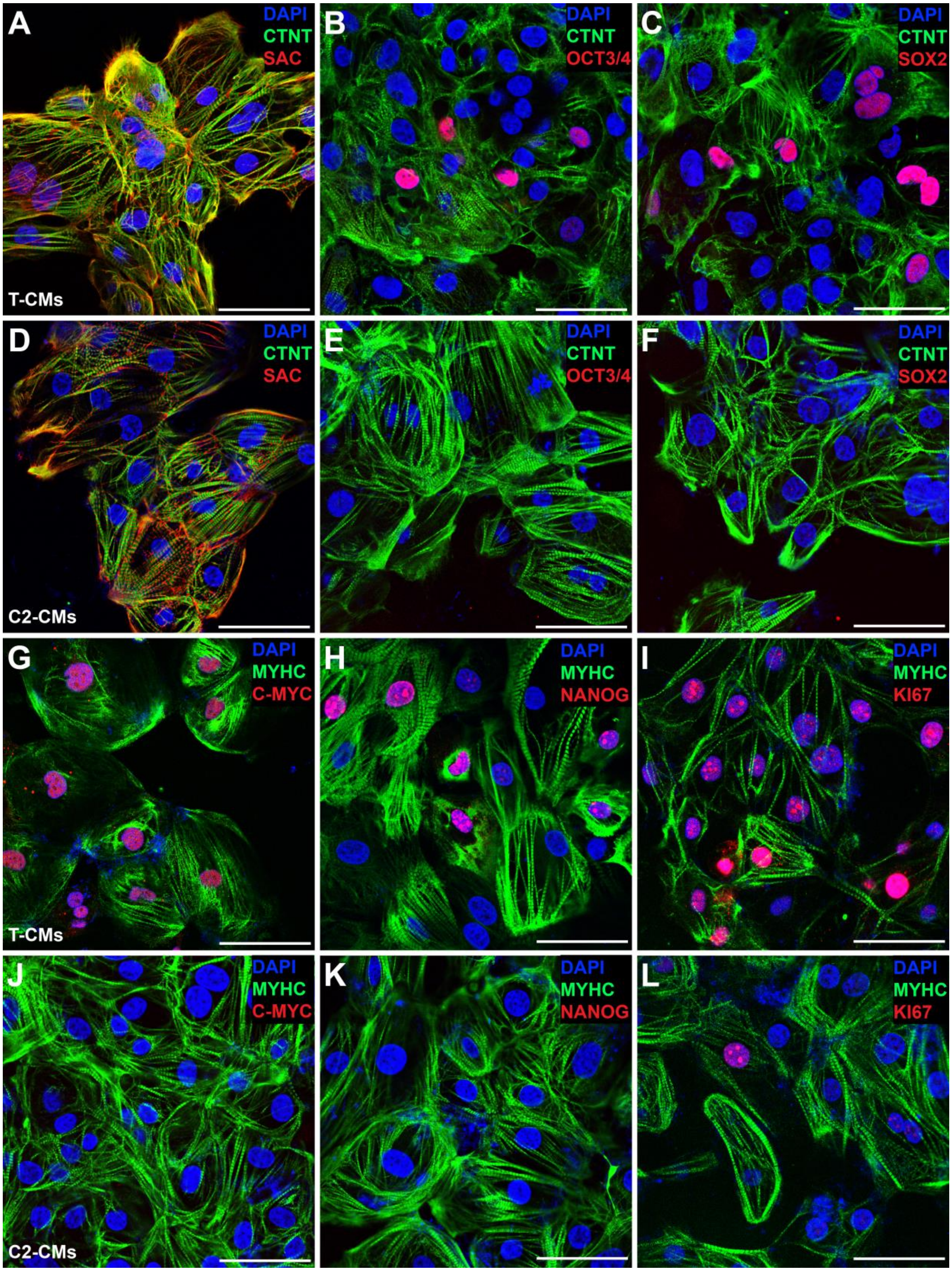

Supplemental Figure S1

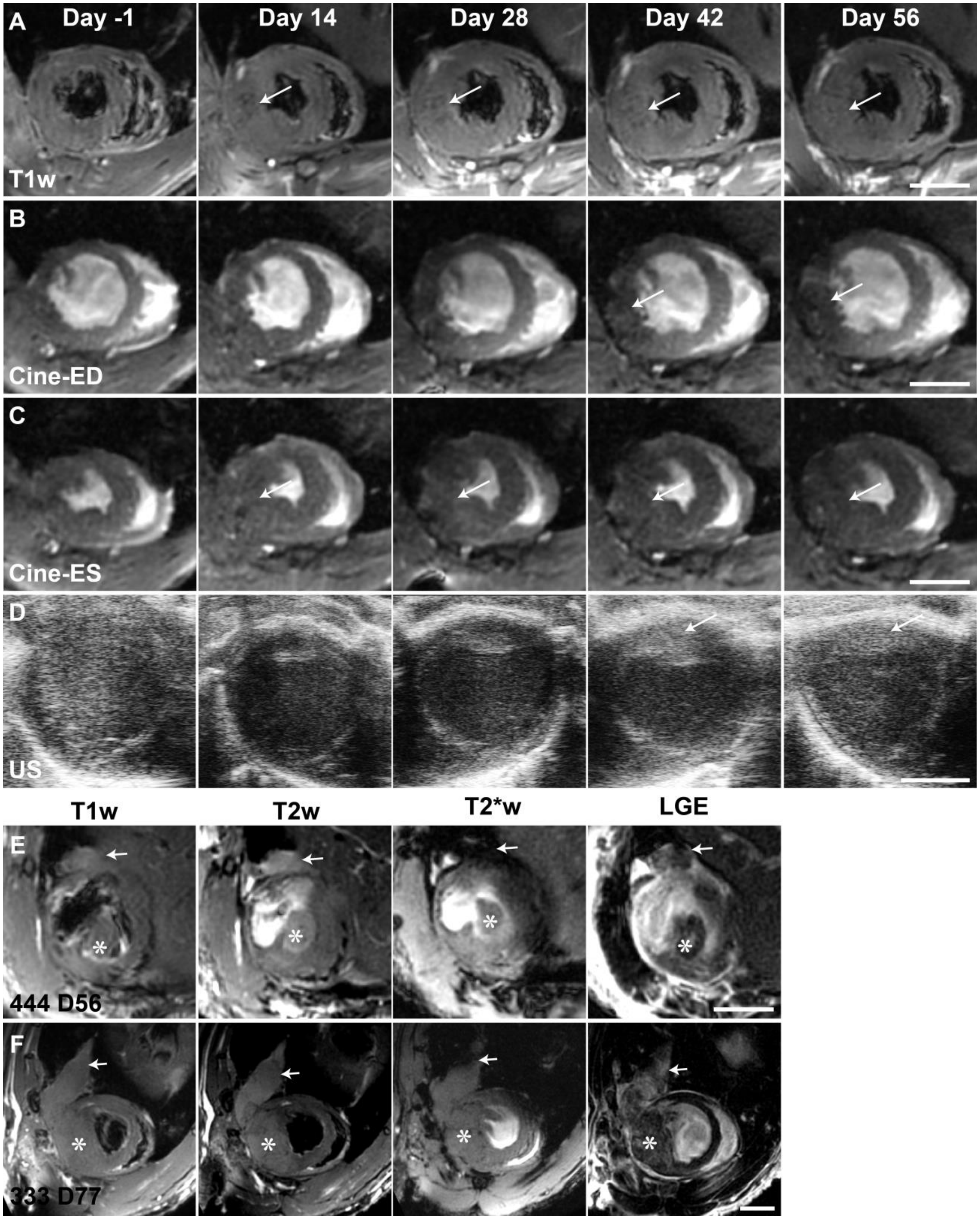

Supplemental Figure S2

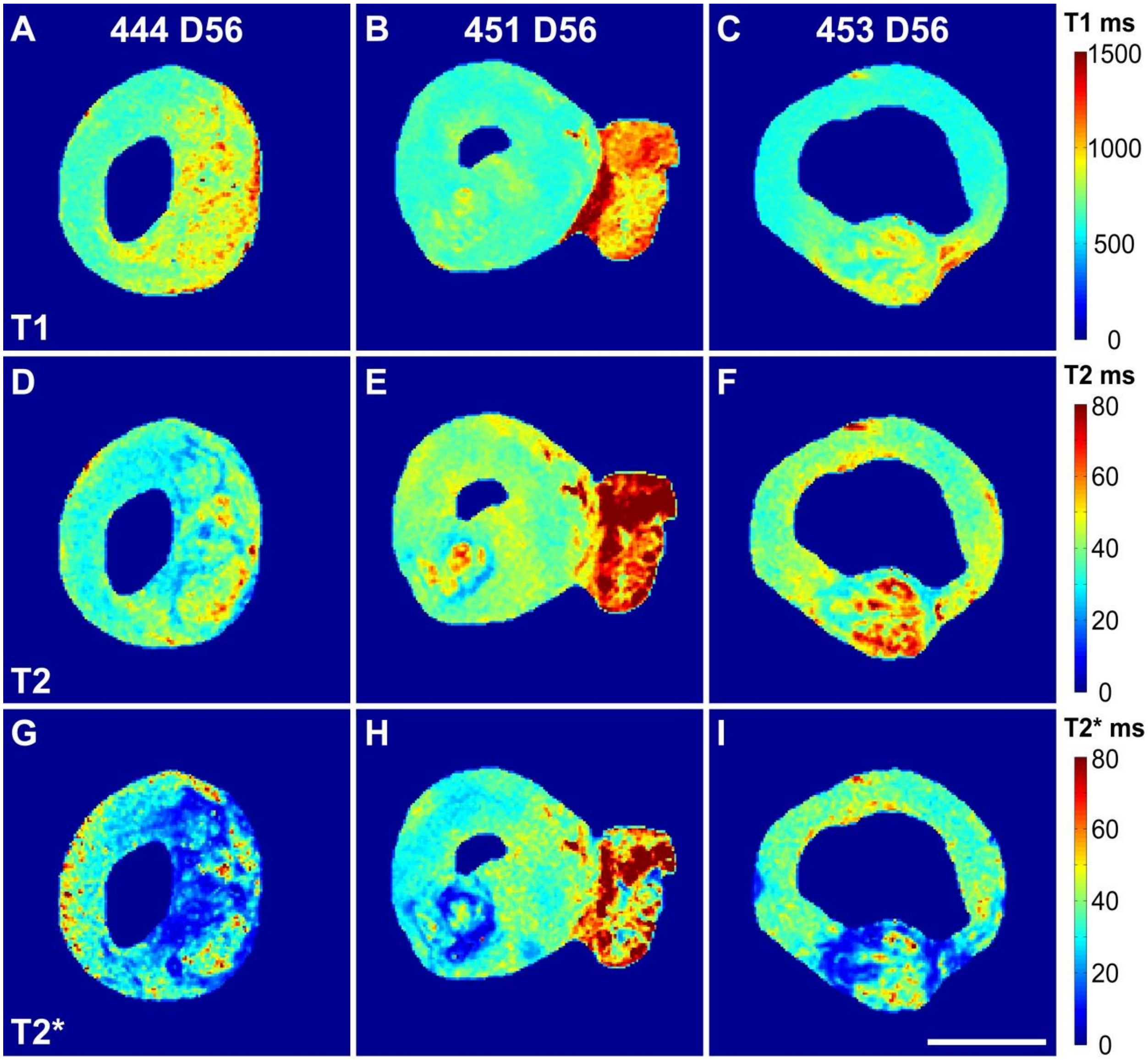

Supplemental Figure S3

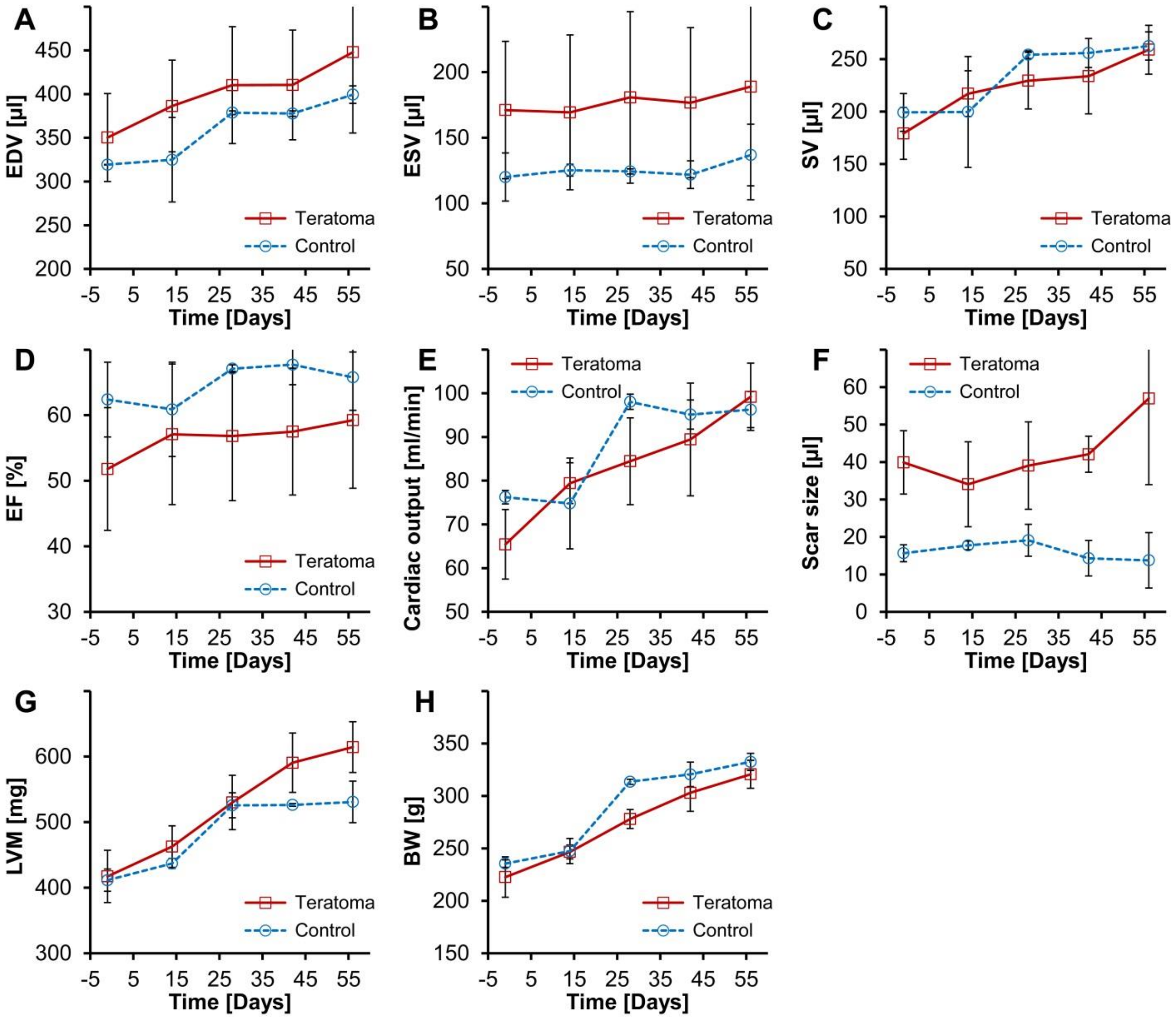

Supplemental Figure S4

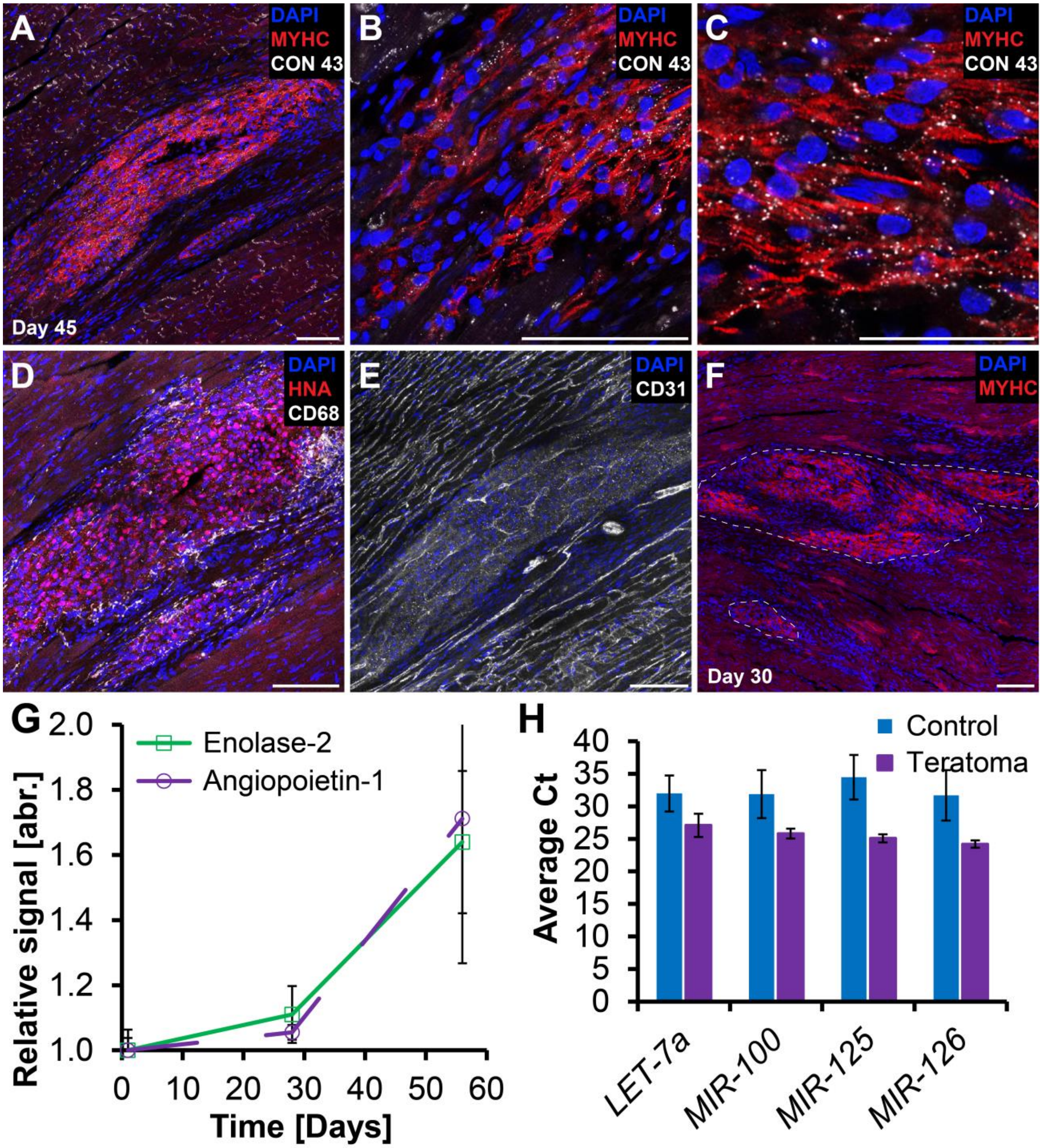

Supplemental Figure S5

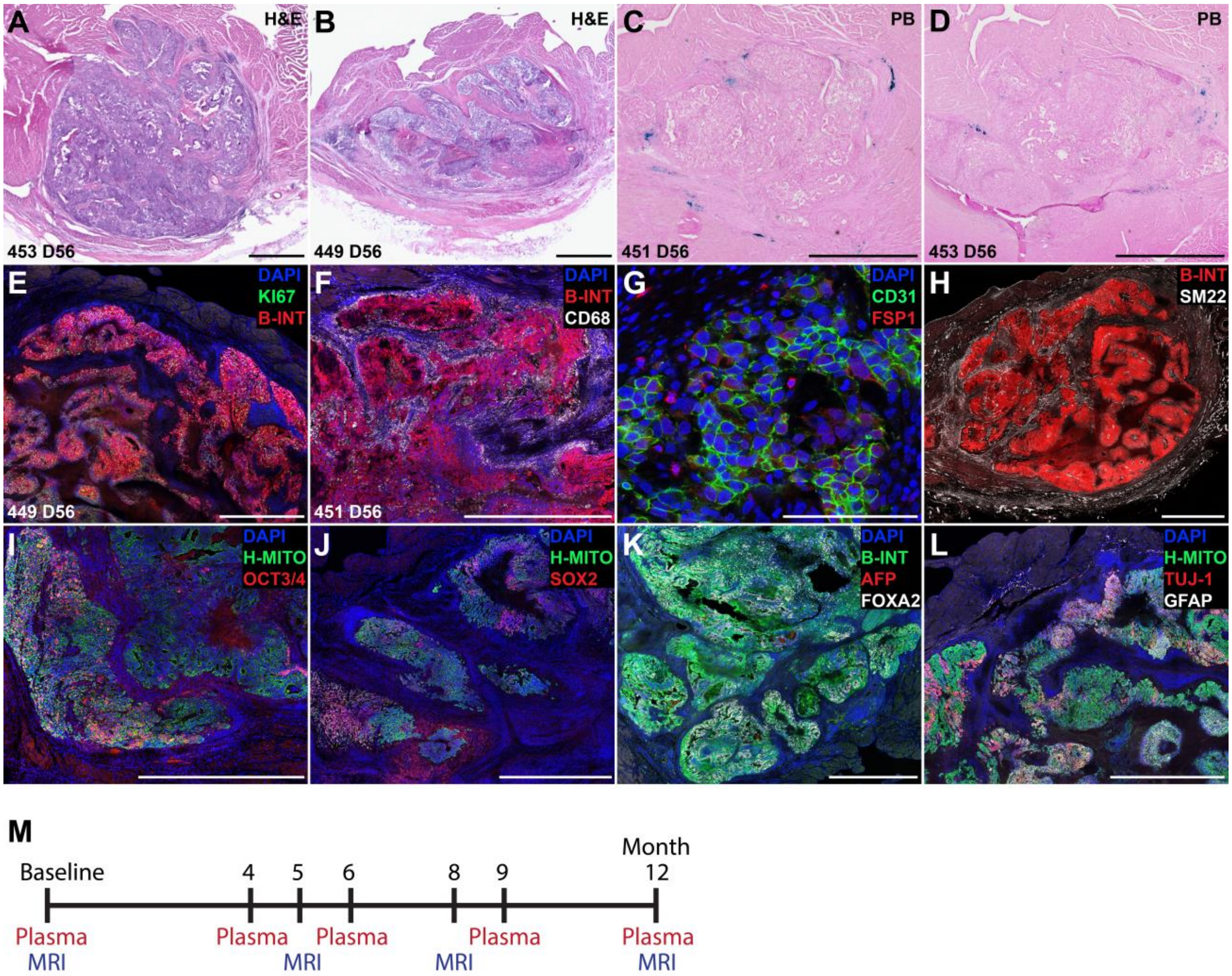

Supplemental Figure S6

SUPPLEMENTAL FIGURE LEGENDS

Supplemental Figure S1: Lentiviral-derived iPSC-CMs showed continued expression of pluripotency markers in contrast to Sendai virus-derived iPSC-CMs. (A-C) Human CMs from lentiviral iPSC line T showed normal sarcomeric structures (cardiac troponin T: CTNT; sarcomeric alpha actinin: SAC), but continued to express OCT3/4 and SOX2. **(D-F)** CMs from control iPSCs (C2) which were generated via a non-integrating method (Sendai virus) displayed normal sarcomeric structures and stained negative for pluripotency markers OCT3/4 and SOX2. **(G-I)** Almost all CMs from the lentiviral line T stained positive for the cell cycle marker C-MYC and about half of the CMs stained positive for NANOG. CMs from this cell line were highly proliferative with all nuclei staining positive for KI67. **(J-L)** CMs from a Sendai virus reprogrammed control iPSC line (C2) did not contain any C-MYC or NANOG positive cells. Only a small number of CM nuclei stained positive for KI67 indicating limited proliferation (representative images from two independent differentiation lots). Scale bars: 50 µm.

Supplemental Figure S2: Serial MRI using T_1w and cine acquisitions as well as ultrasound depicting teratoma growth. (A) Representative T_1-weighted (T1w) images of a rat heart one day before and 2, 4, 6, and 8 weeks after cell delivery. T_1 differences between teratoma and normal myocardium were insufficient to generate enough contrast. The teratoma could only be detected indirectly via increased myocardial wall thickness (white arrows are pointing to teratomas). **(B,C)** End-diastolic (ED) and end-systolic (ES) cine images from the same animal. Similar to T_1w images, teratomas could only be detected indirectly via increased myocardial wall thickness. **(D)** Teratomas could be detected via increased myocardial wall thickness on ultrasound images once they had reached a size of >50 mm^3 (white arrows are pointing to teratomas). **(E-F)** T_1-weighted (T1w), T_2-weighted (T2w), T_2*-weighted (T2*w), and late gadolinium enhancement (LGE) images from two teratomas at 56 and 77 days that had spread to the surrounding lung tissue. This spread to the lung could be easily detected on T1w, T2w as well as LGE images. Scale bars: 5 mm.

Supplemental Figure S3: T_1-, T_2- and T_2*-maps for hearts with teratoma. (A-C) T_1 maps for hearts with a teratoma two month after cell delivery (n=3 rats). Teratomas in the heart had slightly longer relaxation times (801±60 ms) compared to normal myocardium (632±31 ms). **(D-F)** T_2 maps of the same hearts depicted in top row (n=3 rats). The core of teratomas had longer relaxation times (49±8 ms) and was surrounded by a rim with short relaxation times (30±3 ms) compared to normal myocardium (38±2 ms). **(G,I)** While T_2* maps (n=3 rats) showed much lower relaxation times (14±1 ms) at the teratoma rim, relaxation times in the core (36±2 ms) were similar to normal myocardium (35±5 ms). Scale bar: 5 mm.

Supplemental Figure S4: Teratoma growth had no significant effect on cardiac remodeling. (A-E) We did not observe any statistically significant differences between hearts with cell engraftment and teratoma growth (n=5) and hearts without cell engraftment and teratoma growth (Control, n=3). As a general trend, ejection fraction (EF) did increase slightly in both groups with no significant differences between them (P=0.72). **(F)** Scar volumes for hearts with teratoma growth were higher before cell injection, but there was a significant difference in the change of scar volume over time (P=0.05). Control animals showed a small decline in scar volume while teratoma hearts showed an increase in scar volume, particularly from day 28 onwards (teratoma masses were classified as scar). **(G)** Similar to the increases in scar size from

day 28 onwards, left ventricular mass (LVM) was similar between both groups but started to diverge after day 28. This led to a difference in LVM over time, albeit not statistically significant (P=0.06). **(H)** The body weight (BW) for both groups increased over time with similar rates (P=0.48). EDV: end-diastolic volume, ESV: end-systolic volume, SV: stroke volume, CO: cardiac output (Data: mean ± standard deviation).

Supplemental Figure S5: Engraftment of human cardiomyocytes in control hearts. (A) Injection of H7-CMs led to the formation of grafts consisting primarily of human cardiomyocytes (human β-myosin heavy chain: MYHC) at 45 days after cell delivery. **(B)** Human cardiomyocytes started to align themselves to rat cardiomyocytes at the edges of grafts. **(C)** Expression of the gap junction protein Connexin 43 (CON 43) was low indicating the immaturity of these cells. **(D)** Human grafts (human nuclear antigen: HNA) contained few macrophages (CD68), indicating stable engraftment. **(E)** Grafts were vascularized by host-derived blood vessels (CD31). **(F)** Human cardiomyocyte grafts were typically 300-400 µm in thickness and spanned 1-2 mm along the circumference of the rat heart (representative images from one of 4 analyzed rat hearts). **(G)** Protein arrays performed on plasma from an independent cohort of rats (4 animals with confirmed teratoma included) identified enolase-2 and angiopoietin-1 as potential protein based biomarkers. **(H)** Plasma samples from the same animals were used to screen for microRNA based biomarkers. Four microRNAs with statistically significant increases in plasma concentration (*LET-7A, MIR-100, MIR-125, and MIR-126*; P<0.05) were identified. Scale bars: A,B,D,E,F: 100 µm, C: 50 µm.

Supplemental Figure S6: Teratomas were highly proliferative, surrounded by an iron rich rim, and contained cells from all three germ layers. (A,B) H&E images showing two teratomas 56 days after cell transplantation (n=2 representative of five analyzed rat hearts). Most teratomas contained primarily endoderm, some mesoderm and undifferentiated cells with necrosis in the core (n=5 rats). Extensive formation of glands with endodermal appearance was also observed. The teratoma from animal #449 (B) contained primarily undifferentiated cells, some mesoderm and a necrotic core. **(C,D)** Prussian blue (PB) staining confirmed the presence of iron rich cells in the teratoma rim. These cells were likely responsible for the short T_2 and T_2* relaxation times observed on the edges of teratomas. **(E)** A substantial number of human cells (human β1 Integrin: B-INT) were in an active state of the cell cycle (KI67) in line with the proliferative nature of these teratomas. **(F)** Chronic inflammation with macrophages (CD68) along the rim and few macrophages in human cell clusters was observed. **(G)** Teratomas contained cells staining positive for a human specific platelet endothelial cell adhesion molecule marker (CD31). However, these cells did not display endothelial morphology and were likely ESCs. **(H)** Small arteries surrounded by smooth muscle cells (SM22) were abundant in normal myocardium but few were observed in teratomas. SM22 positive, fibroblast-like cells were observed surrounding human cell clusters. We also observed some human cells staining positive for the mesodermal marker SM22. **(I,J)** Teratomas contained human cell clusters (human mitochondria: H-MITO) staining positive for pluripotency markers OCT-3/4 and SOX-2. **(K)** A large number of human cells stained positive for the endodermal transcription factor forkhead box protein A2 (FOXA2). Gland-like structures containing α-fetoprotein (AFP) positive cells (endoderm) were found in most teratomas. **(L)** Small cell clusters staining positive for the ectodermal markers neuron-specific class III beta-tubulin (TUJ-1) and glial fibrillary acidic protein (GFAP) were observed in most teratomas. **(M)** The hypothetical spacing for plasma

collection and MRI is based on the assumption of linear scalability for the detection sensitivity according to differences in plasma volume and imaging resolution between rats and humans. Contamination of a cell product by undifferentiated cells bellow the detection threshold of current assays was assumed, which leads to a long lag time before human detection sensitives are reached. Furthermore, the highest teratoma growth rate reported for humans in the literature was used to estimate teratoma growth. Scale bars: A,B,C,D,E,F,H,I,J,K,L: 1000 µm, G: 100 µm.

SUPPLEMENTAL TABLES

Supplemental Table 1: Cardiac differentiation yields

(n=3)	CTNT positive	CTNT negative
C1-CMs	79 ± 1	21 ± 1
C2-CMs	82 ± 3	18 ± 3
T-CMs	86 ± 2	14 ± 2

Supplemental Table 2: Relaxation parameters

(n=3)	Remote	Teratoma core	Teratoma margin
T_1 [ms]	632 ± 31	801 ± 60	
T_2 [ms]	38 ± 2	49 ± 8	30 ± 3
T_2^* [ms]	36 ± 2	35 ± 5	14 ± 1

Supplemental Table 3: MRI measurements for control and teratoma groups

	Day -1		Day 56	
	Control (n=3)	Teratoma (n=5)	Control (n=3)	Teratoma (n=5)
Body weight [g]	236 ± 10	223 ± 19	333 ± 8	321 ± 13
Heart rate [bmp]	384 ± 27	367 ± 27	367 ± 7	384 ± 17
EDV [µl]	319.3 ± 5.3	350.3 ± 50.3	399.4 ± 10.6	447.9 ± 92.5
ESV [µl]	120.1 ± 18.3	171.2 ± 52.4	136.9 ± 23.5	189.0 ± 86.2
SV [µl]	199.2 ± 18.0	179.1 ± 24.7	262.5 ± 13.5	258.9 ± 23.2
EF [%]	62.4 ± 5.7	51.8 ± 9.3	65.8 ± 5.0	59.2 ± 10.4
CO [ml/min]	76.2 ± 1.5	65.4 ± 8.0	96.3 ± 4.1	99.2 ± 7.7
LVM [mg]	411.5 ± 16.8	417.3 ± 39.8	530.8 ± 31.6	614.4 ± 38.7
Scar [µl]	15.7 ± 2.3	39.9 ± 8.4	13.8 ± 7.4	57.0 ± 23.0
Infarct [% of LVM]	4.0 ± 0.7	10.0 ± 1.6	2.7 ± 1.3	9.6 ± 3.4

Supplemental Table 4: MRI measurements for C1-CM, PBS and T-CM treatment groups

	Day -1			Day 28		
	C1-CMs (n=12)	PBS (n=12)	Teratoma (n=10)	C1-CMs (n=12)	PBS (n=12)	Teratoma (n=10)
Body weight [g]	247 ± 15	253 ± 23	222 ± 17	310 ± 17	296 ± 27	261 ± 44
Heart rate [bmp]	406 ± 26	405 ± 22	378 ± 23	375 ± 24	377 ± 29	376 ± 27
EDV [µl]	375 ± 51	396 ± 47	370 ± 45	466 ± 45	497 ± 67	438 ± 77
ESV [µl]	167 ± 33	181 ± 32	179 ± 42	183 ± 33	216 ± 61	194 ± 55
SV [µl]	209 ± 35	216 ± 27	192 ± 30	283 ± 24	281 ± 48	245 ± 45
EF [%]	56 ± 6	55 ± 5	52 ± 8	61 ± 4	57 ± 8	56 ± 8
CO [ml/min]	84 ± 14	87 ± 10	72 ± 12	106 ± 11	106 ± 18	92 ± 15
LVM [mg]	362 ± 28	393 ± 59	385 ± 77	454 ± 65	434 ± 56	456 ± 71
Scar [µl]	45 ± 17	58 ± 18	48 ± 12	46 ± 14	47 ± 17	44 ± 13
Infarct [%LVM]	13 ± 4	15 ± 3	10 ± 2	11 ± 2	11 ± 4	9 ± 2

Supplemental Table 5: List of antibodies used for this study

**Antibody**	**Dilution**	**Vendor**	**Order Nr.**
Donkey secondary antibodies	1:200	Jackson ImmunoResearch	
human cardiac Troponin T (CTNT)	1:200	Abcam	ab45932
human CD31 (PECAM1)	1:100	Dako	M082329-2
human Mitochondria (H-MITO)	1:200	Millipore	MAB1273A4
human Nuclear Antigen (HNA)	1:100	Millipore	MAB1281
human Anti-Integrin β1 (CD29)	1:200	Millipore	MAB1965
human β Myosin heavy chain A4.951	1:100	Dev. Studies hybridoma bank	A4.951
FSP1/S100A4	1:200	Millipore	07-2274
Connexin 43 (CON 43)	1:300	Sigma	C6219
CD31	1:100	Millipore	04-1074
sarcomeric alpha actinin (SAC)	1:100	Sigma	A7811-.2ML
CD68	1:300	Abcam	ab125212
SM22	1:300	Abcam	ab14103
KI67	1:300	Millipore	AB92601
CD90	1:200	Abcam	ab92574
SOX2	1:100	R&D Systems	AF4666
OCT3/4	1:50	BD Biosciences	611203
C-MYC	1:50	Millipore	06-340
NANOG	1:100	eBioscience	14-5768-82
SSEA4	1:200	Abcam	ab16287
β III Tubulin (TUJ-1)	1:100	Abcam	ab14545
HNF-3 β/FOXA2	1:100	R&D Systems	AF2400
α-Fetoprotein (AFP)	1:100	Dako	A0008
Glial fibrillary Acidic Protein (GFAP)	1:100	Dako	Z0334

SUPPLEMENTAL EXPERIMENTAL PROCEDURES

Derivation of human iPSCs

Informed consent was acquired from a patient with dilated cardiomyopathy to collect skin fibroblasts and generate induced pluripotent stem cells (iPSC). Skin fibroblasts were reprogrammed to iPSCs using lentivirus as previously described (Sun et al., 2009). A clone which continued to express reprogramming factors at high levels after differentiating (line T) as well as a clone which did not (line C1) were selected. Alternatively, generation of iPSCs with integration-free Sendai-virus methods (line C2) was performed as previously described (Ebert et al., 2014). Human iPSCs were cultured on Matrigel-coated plates (ES qualified, BD Biosciences, San Diego) using chemically defined E8 medium as previously described (Chen et al., 2011). Cells were passaged using Accutase (Global Cell Solutions) every four days.

Cardiac differentiation

Human iPSCs (lines, T, C1, C2), or H7 ESCs obtained from WiCell (Madison, WI) were grown to 90% confluence. A small molecule-based monolayer technique was employed subsequently for differentiation of human iPSCs or ESCs into beating cardiomyocytes (Ebert et al., 2014; Lian et al., 2012).

Flow cytometry analysis of cardiac markers

CMs were detached as described above and subsequently fixed and permeabilized using BD Cytofix/Cytoperm and BD Perm/Wash kits (BD Biosciences) according to the manufacturer´s instructions. Cells were incubated with a primary mouse anti-cardiac troponin T antibody (CTNT, Thermo Scientific) for 2 hours at 4°C. Afterwards, cells were washed and incubated with a secondary goat anti-mouse IgG (H+L) Alexa 488 antibody (Life Technologies) for 45 min at 4 °C. Cells were washed and analyzed by fluorescent activated cell sorting (FACS; BD Aria II). Data analysis was performed using FlowJo software.

Pluripotency marker analysis

Quantitative real-time PCR (qRT-PCR) for pluripotency markers was performed as described earlier using TaqMan probes (Applied Biosystems / Life Technologies) for *CMYC*, *OCT4*, and *KLF4* (Lan et al., 2013).

Karyotyping

The G-band or GTW banding karyotype at >400 band resolution was performed by the Stanford Cytogenetics Laboratory as described previously (Lan et al., 2013).

Myocardial infarction and cell transplantation

All animal procedures were approved by the ethics committee of Stanford University School of Medicine. Male nude rats (Crl:NIH-Foxn1rnu, n=79) (Charles River Laboratories, Wilmington, MA) aged 8-10 weeks were used for this study. Anesthesia was induced with 3-4% isoflurane in oxygen and was maintained at 1.5-2%. Rats were placed on a heating pad with a feedback controller and maintained at 36±1 °C during the surgical procedure. Pre-emptive analgesia in the form of buprenorphine 0.01-0.05 mg/kg or Carprofen subcutaneous and bupivacaine 0.5-2 mg/kg (local infiltration) was provided as well as eye lubrication ointment. After intubation, rats were ventilated and left thoracotomies were performed between the 3rd and 4th ribs. The pericardial sac was opened and the left anterior descending (LAD) coronary artery was occluded for 1 hour with

a 5-0 Prolene suture close to the left atrial appendage. The chest and the incision were closed after LAD reperfusion. Initial MRI scans were performed 3 days after MI surgery to exclude rats with insufficient infarcts (EF >65%, baseline EF before surgery was 73±1%). All rats fulfilling the inclusion criteria (65/79) were used for cell injections. Four days after MI (one day after initial MRI), a second thoracotomy was performed and $1x10^7$ human CMs or PBSs was injected at three injection sites around the scar area (15 µl per injection site). The following cell and PBS injections were used for different aspects of the study; teratoma detection and growth: T-CMs (n=8), alternative biomarkers: T-CMs (n=8), teratoma detection via LGE: T-CMs (n=16), control for assessment of cardiac function: PBS (n=12), changes in cardiac function due to human CM engraftment: C1-CMs (n=12), and biomarker control group for stable human CM grafts: H7-CMs (n=9). All surgeries were performed by an experienced micro-surgeon (M.W.). Rats received postoperative analgesia for three days and antibiotics via their drinking water (Enrofloxacin, 2.5-4 mg/kg) starting with the first surgery.

***In vivo* magnetic resonance imaging (MRI)**

Imaging was performed 1 day prior and every other week for 2 month after cell delivery, using a preclinical 7T (MR901 Discovery) horizontal bore scanner (Agilent, Santa Clara, CA) with a shielded gradient system (600 mT/m). Rats were anesthetized with isoflurane (3%) and placed onto an animal cradle in prone position. Animals were kept at 37±0.4 °C (during image acquisition) via an air heating system while oxygen and anesthetics (1-2% isoflurane) were supplied via a nose cone (0.5 L/min). Data acquisition was performed with a 4-channel phased array receive only surface coil (Rapid MR International, Columbus, OH) placed around the chest and centered in a decoupled 72 mm transmit/receive volume coil (Agilent). Long- and short-axis scout images were acquired to define the two- and four-chamber long-axis views. The cine long-axis views were used to define the short-axis orientation. A prospectively double gated (ECG and respiration) spoiled gradient echo sequence was used to acquire cine cardiac images with the following parameters for standard cine acquisitions: TE 1.5 ms, TR 6-8 ms, flip angle 15°, slice thickness 1 mm, no slice separation, FOV 50×50 mm², matrix size 192×192, NSA 1 for short-axis and 2 for long-axis. Twenty cine-frames were recorded to cover the cardiac cycle. A single short-axis slice was obtained in approximately 45 seconds, leading to a total scan time of 11-13 min covering the heart from base to apex (14-15 slices). A double gated spin-echo sequence was used for T_1-weighted acquisitions using the following imaging parameters: TE 9.7 ms, TR 700 ms, slice thickness 1 mm, no slice separation, FOV 40×40 mm², matrix size 192×192, NSA 4. For T_2-weighted acquisitions, a double gated spin echo sequence with the following imaging parameters was used: TE 20 ms, TR 700 ms, slice thickness 1 mm, no slice separation, FOV 40×40 mm², matrix size 192×192, NSA 4. A fast spoiled gradient echo sequence with the following imaging parameters was used to acquire T_2*-weighted images: TE 7 ms, TR 10.3 ms, flip angle 20°, slice thickness 1 mm, FOV 40×40 mm², matrix size 192×192, NSA 3. After cine, T_1-weighted, T_2-weighted and T_2*-weighted acquisitions, gadolinium was injected via an i.v. infusion line (0.8 mmol/kg, Magnevist Bayer, Germany). Late gadolinium enhancement (LGE) images were acquired 8 min after i.v. infusion using an inversion recovery gradient echo sequence with inversion time optimized to null the signal from healthy myocardium (Price et al., 2011). Imaging parameters for these acquisitions were as follows: TE 1.4 ms, TR one breathing interval, TI 280-370 ms, flip angle 90°, slice thickness 1 mm, no slice separation, FOV 40×40 mm², matrix size 192×192, NSA 2, views per segment 2. The acquisition time was roughly 1 min per slice. The imaging protocol for one rat typically required 70 min.

Ex vivo MRI

Rat hearts were fixed in 4% PFA over night at 4 °C. Following fixation, hearts were embedded in 2% low melting point agarose in 50 ml tubes. Imaging was performed using a preclinical 7T horizontal bore scanner with a shielded gradient system and a 30 mm inner diameter Millipede coil (Agilent, Santa Clara, CA). For T_1 mapping, a series of multi-slice spin echo images was acquired using the following imaging parameters: TE 8 ms, TR 4000 ms, TI 50, 100, 200, 300, 400, 800 ms, slice thickness 0.5 mm, 0.5 mm gap, FOV 25×25 mm², matrix size 256×256, NSA 1. For T_2 mapping, a series of multi-slice spin echo images was acquired using the following imaging parameters: TE 10, 15, 20, 30, 40, 76 ms, TR 4000 ms, slice thickness 0.5 mm, 0.5 mm gap, FOV 25×25 mm², matrix size 256×256, NSA 1. For T_2* mapping, a series of spoiled gradient echo images was acquired using the following imaging parameters: TE 5, 10, 15, 18 ms, TR 4000 ms, flip angle 40°, slice thickness 0.5 mm, 0.5 mm gap, FOV 25×25 mm², matrix size 256×256, NSA 1. T_1, T_2 and T_2* maps were generated by fitting the spin echo and gradient echo signal equations using a custom Matlab script. Free hand regions of interest were drawn to estimate average relaxation parameters for remote myocardium, center of teratoma and teratoma rim.

MRI data analysis

For the assessment of cardiac function, cine images from one animal were combined to a dataset, randomized, and anonymized. Data analysis was performed using the semi-automatic segmentation software Segment (Medviso AB, Sweden) (Heiberg et al., 2010) as previously described (Riegler et al., 2010). For the detection of possible teratomas and the quantification of teratoma volume, acquisitions from weeks 2, 4, 6 and 8 were paired with the pre-scan (day -1) for each animal and blinded. Regions of interest (ROI) were drawn manually by an experienced user outlining suspected teratomas using ImageJ. Cine images were scored as teratoma positive or negative without volumetric quantification.

Ultrasound imaging

B-mode ultrasound imaging was performed at the same days as MRI was performed. Rats were anesthetized using 2% isoflurane and imaged in a supine position on a heated platform. Imaging was performed using a real-time microvisualization transducer (MS250) with a frequency of 25 MHz connected to a Vevo 2100 ultrasound system (Visualsonics, Toronto, Canada). Three to four B-mode cine images were acquired in long-axis orientation and four to five in short-axis orientation. Acquired data was scored by a blinded observer for visible signs of teratoma.

Immunodetection and histological methods

Heart fixation and section preparation: Rats were anesthetized, 1 ml of heparin (1000 Units, APP Pharmaceuticals, IL) was injected i.v, the chest was opened, a 25G butterfly needle inserted into the left ventricle via the apex, and 6 ml of blood were collected in EDTA tubes. Following blood collections, cuts into the liver were made and their hearts were perfused with 50 ml cold PBS (4 ºC) containing 0.1 mol/l KCl. Hearts were excised and fixed over-night at 4 ºC in PBS with 4% PFA. Following fixation, hearts were transferred into 30% sucrose solution and kept at 4 ºC until equilibrium was reached. Hearts were embedded in OCT and frozen in hexane containing dry ice. Sections were cut with a cryostat (Leica, Wetzlar, Germany) collected on glass slides, dried and stored at -80 ºC.

Preparation of cell samples: Human iPSCs or iPSC-CMs were seeded in chambered coverslides (Lab-Tek, Nalge Nuc International, NY) and cultured for 3-4 days. Cells were washed once with PBS before fixation with 4% PFA for 10 min at room temperature. After fixation, cells were permeabilized for 15 min with PBS containing 0.1% Triton-X at room temperature. Immunostaining was performed as outlined below.

Immunofluorescence: Sections were equilibrated to room temperature, washed 3x 10 min with PBS, permeabilized with 0.5% Triton-X (Sigma) in PBS for 60 min at room temperature followed by incubation in blocking solution (5% donkey serum in PBS + 0.1% Tween20, Sigma) for 60 min. Sections were incubated with primary antibodies (see Supplemental Table S5), and diluted in blocking solution over night at 4 ºC in a humid chamber. After washing 3x 15 min with PBS + 0.1% Tween20, sections were incubated with secondary antibodies diluted in blocking solution for 1 hour followed by a final washing step (3x 15 min in PBS + 0.1% Tween20) and covered with cover slips using self-hardening mounting media. Confocal microscopy was performed using a Leica SP8 microscope (Leica, Wetzlar, Germany). A series of images was acquired with a 20x or 63x oil immersion objective. Images were stitched together to generate composite images to display the entire cross-section of a teratoma.

Screening for cell engraftment: Hearts were cut from apex to base. All sections were collected and mounted on glass slides. Sections on every third slide, advancing the distance from apex to base by 360 μm, were stained against human integrin β1 and human nuclear antigen. Stained slides were manually screened for human grafts using a fluorescence microscope with a 20x objective (Nikon, Japan).

Quantification of plasma biomarkers

Approximately 2 ml of blood were collected directly after each MRI session via a tail vein catheter using EDTA coated tubes (BD, NJ). The final blood collection was performed after terminal anesthesia as described above. Blood was stored on ice for up to 3 hours before centrifugation at 3000g to separate the plasma. Plasma was aliquoted (100 μl per tube) and stored at -80 °C until specific ELISAs were performed. Plasma samples were diluted 1:1 with the respective sample dilution buffers provided. Two or three wells were used for each sample on each ELISA plate. The following ELISA kits were acquired and performed following manufacturers protocols: carcinoembryonic antigen (CEA, Abcam: ab99992), α-fetoprotein (AFP, R&D Systems: DAFP00), fibroblast growth factor (FGF, Invitrogen: KHG0021), human chorionic gonadotropin (HCG, Sigma: RAB0092), vascular endothelial growth factor (VEGF, Sigma: RAB0508), lactate dehydrogenase (LDH, Abcam: ab116693), alkaline phosphatase (ALP, Abcam: ab83369 and RayBiotech: ELH-ALKP-1), and cancer antigen-125 (CA-125, Abcam: ab108653). Absorbance was measured at the recommended wavelength for the different ELISAs using a plate reader (GloMax-Multi, Promega, WI).

RNA Extraction and cDNA Synthesis

Total RNA, including miRNA, was extracted from 150 µl serum by using a miRNeasy Serum/Plasma Kit (Qiagen) according to the manufacturer's protocol. Briefly, five volumes of QIAzol lysis reagent along with a synthetic spike-in control, *Caenorhabditis elegans* miR-39 (1.6 × 108 copies/µl), was added to the lysed samples for internal normalization. After adding an equal volume of chloroform, the samples were centrifuged for 15 min at 12,000 g at 4°C. The upper aqueous phase was mixed with 1.5 volumes of 100% ethanol, transferred to a spin column, centrifuged, washed, and eluted in 14 µl RNase-free water. 2 µl of each RNA sample was used

for cDNA synthesis using the miScript II RT kit (QIAGEN). The reaction mixture was incubated for 60 min at 37°C, followed by denaturation for 5 min at 95°C. Each cDNA was further diluted to 200 µl with RNase-free water and stored at -20°C until use.

microRNA Screening

Screening was performed using the Human Cancer Pathway Finder miRNA PCR array (Qiagen) according to the manufacturer's protocol. All the miRNAs included in the arrays have previously been reported to be implicated in various forms of cancers. Each array included C. elegans miR-39 primer assays for internal normalization, positive PCR control (PPC) assays, and miRNA reverse transcription control (miRTC) assays. The data sets were calibrated using a C. elegans *miR-39* assay, which detected the spike-in control that was added to the serum samples during RNA extraction. An Excel-based miRNA PCR Array Data Analysis tool (Qiagen; http://pcrdataanalysis.sabiosciences.com/mirna/arrayanalysis.php) was used for data analysis.

Proteome profiler array

Screening was performed using the Human XL Oncology Array Kit array (R&D Systems: ARY026) according to the manufacturer's protocol using 500 µl of plasma for each time point.

SUPPLEMENTAL REFERENCES

Chen, G., Gulbranson, D.R., Hou, Z., Bolin, J.M., Ruotti, V., Probasco, M.D., Smuga-Otto, K., Howden, S.E., Diol, N.R., Propson, N.E., *et al.* (2011). Chemically defined conditions for human iPSC derivation and culture. Nat Methods *8*, 424-429.

Ebert, A.D., Kodo, K., Liang, P., Wu, H., Huber, B.C., Riegler, J., Churko, J., Lee, J., de Almeida, P., Lan, F., *et al.* (2014). Characterization of the molecular mechanisms underlying increased ischemic damage in the aldehyde dehydrogenase 2 genetic polymorphism using a human induced pluripotent stem cell model system. Sci Transl Med *6*, 255ra130.

Heiberg, E., Sjogren, J., Ugander, M., Carlsson, M., Engblom, H., and Arheden, H. (2010). Design and validation of Segment--freely available software for cardiovascular image analysis. BMC Med Imaging *10*, 1.

Lan, F., Lee, A.S., Liang, P., Sanchez-Freire, V., Nguyen, P.K., Wang, L., Han, L., Yen, M., Wang, Y., Sun, N., *et al.* (2013). Abnormal calcium handling properties underlie familial hypertrophic cardiomyopathy pathology in patient-specific induced pluripotent stem cells. Cell Stem Cell *12*, 101-113.

Lian, X., Hsiao, C., Wilson, G., Zhu, K., Hazeltine, L.B., Azarin, S.M., Raval, K.K., Zhang, J., Kamp, T.J., and Palecek, S.P. (2012). Robust cardiomyocyte differentiation from human pluripotent stem cells via temporal modulation of canonical Wnt signaling. Proc Natl Acad Sci U S A *109*, E1848-1857.

Price, A.N., Cheung, K.K., Lim, S.Y., Yellon, D.M., Hausenloy, D.J., and Lythgoe, M.F. (2011). Rapid assessment of myocardial infarct size in rodents using multi-slice inversion recovery late gadolinium enhancement CMR at 9.4T. J Cardiovasc Magn Reson *13*, 44.

Riegler, J., Cheung, K.K., Man, Y.F., Cleary, J.O., Price, A.N., and Lythgoe, M.F. (2010). Comparison of segmentation methods for MRI measurement of cardiac function in rats. J Magn Reson Imaging *32*, 869-877.

Sun, N., Panetta, N.J., Gupta, D.M., Wilson, K.D., Lee, A., Jia, F., Hu, S., Cherry, A.M., Robbins, R.C., Longaker, M.T., *et al.* (2009). Feeder-free derivation of induced pluripotent stem cells from adult human adipose stem cells. Proc Natl Acad Sci U S A *106*, 15720-15725.
